# Absence of miRNA-146a Differentially Alters Microglia Function and Proteome

**DOI:** 10.3389/fimmu.2020.01110

**Published:** 2020-06-05

**Authors:** Nellie A. Martin, Kirsten H. Hyrlov, Maria L. Elkjaer, Eva K. Thygesen, Agnieszka Wlodarczyk, Kirstine J. Elbaek, Christopher Aboo, Justyna Okarmus, Eirikur Benedikz, Richard Reynolds, Zoltan Hegedus, Allan Stensballe, Åsa Fex Svenningsen, Trevor Owens, Zsolt Illes

**Affiliations:** ^1^Department of Neurology, Odense University Hospital, Odense, Denmark; ^2^Department of Neurobiology Research, Institute of Molecular Medicine, University of Southern Denmark, Odense, Denmark; ^3^Institute of Clinical Research, BRIDGE, University of Southern Denmark, Odense, Denmark; ^4^Department of Health Science and Technology, Aalborg University, Aalborg, Denmark; ^5^Sino-Danish Center for Education and Research, University of Chinese Academy of Sciences, Beijing, China; ^6^Department of Brain Sciences, Imperial College London, London, United Kingdom; ^7^Laboratory of Bioinformatics, Biological Research Centre, Szeged, Hungary; ^8^Department of Biochemistry and Medical Chemistry, University of Pecs, Pecs, Hungary

**Keywords:** miR-146a, microglia, cuprizone, multiple sclerosis lesion, proteome, phagocytosis, migration, CD11c

## Abstract

**Background:** MiR-146a is an important regulator of innate inflammatory responses and is also implicated in cell death and survival.

**Methods:** By sorting CNS resident cells, microglia were the main cellular source of miR-146a. Therefore, we investigated microglia function and phenotype in miR-146a knock-out (KO) mice, analyzed the proteome of KO and wild-type (WT) microglia by LC-MS/MS, and examined miR-146a expression in different brain lesions of patients with multiple sclerosis (MS).

**Results:** When stimulated with LPS or myelin *in vitro*, microglia from KO mice expressed higher levels of IL-1β, TNF, IL-6, IL-10, CCL3, and CCL2 compared to WT. Stimulation increased migration and phagocytosis of WT but not KO microglia. CD11c^+^ microglia were induced by cuprizone (CPZ) in the WT mice but less in the KO. The proteome of *ex vivo* microglia was not different in miR-146a KO compared to WT mice, but CPZ treatment induced differential and reduced protein responses in the KO: GOT1, COX5b, CRYL1, and cystatin-C were specifically changed in KO microglia. We explored discriminative features of microglia proteomes: sparse Partial Least Squares-Discriminant Analysis showed the best discrimination when control and CPZ-treated conditions were compared. Cluster of ten proteins separated WT and miR-146a KO microglia after CPZ: among them were sensomes allowing to perceive the environment, Atp1a3 that belongs to the signature of CD11c^+^ microglia, and proteins related to inflammatory responses (S100A9, Ppm1g). Finally, we examined the expression of miR-146a and its validated target genes in different brain lesions of MS patients. MiR-146 was upregulated in all lesion types, and the highest expression was in active lesions. Nineteen of 88 validated target genes were significantly changed in active lesions, while none were changed in NAWM.

**Conclusion:** Our data indicated that microglia is the major source of miR-146a in the CNS. The absence of miR-146a differentially affected microglia function and proteome, and miR-146a may play an important role in gene regulation of active MS lesions.

## Introduction

Microglia that make up 15–20% of total number of brain cells, are the tissue resident macrophages and the main immune defense of the CNS. Microglia are involved in brain development, neuronal plasticity in adulthood, they respond to injury and participate in repair and clearance of plaques (1). As the CNS resident innate immune cells, microglia play a key role in immune responses. They are antigen presenting cells expressing MCH class I and II, and can secrete both pro-inflammatory cytokines such as IL-1β, and TNF, and anti-inflammatory cytokines such as IL-10. Microglia are also phagocytic cells expressing Fc complement receptors (2). Microglia are activated in response to injury or infection, and similarly to macrophages they express pattern recognition receptors (PRRs) that enable them to sense pathogen associated molecular patterns (PAMPs), and damage associated molecular patterns (DAMPs) such as microbial proteins, saccharides, lipids, RNA and DNA or secretions form damaged neurons and other brain cells (1, 3). Once activated, microglia become phagocytotic and take up cell debris and apoptotic cells. Activated microglia secrete cytokines, migrate toward the injured area, and form a shield around the pathological or injured site (4). There is considerable heterogeneity of microglial activation in the brain, which is dependent on factors in the surrounding micro environment (5). Pro-inflammatory molecules polarize microglia toward an inflammatory phenotype characterized by production/upregulation of IL-1β, IL-6, MHC-II, TNF, iNOS, phagosome acidification, and production of reactive oxygen species (ROS) (6). In contrast, microglia stimulated with cytokines IL-4 and IL-13 produce anti-inflammatory cytokines and neuroprotective factors, express scavenger receptors for phagocytosis, and growth stimulation (3) A CD11c^+^subpopulation of microglia is effective in antigen presentation, but poorly express proinflammatory cytokines (7, 8).

MicroRNAs (miRNAs) are small ~22 nucleotides long non-coding RNAs, that regulate gene expression. They primarily function by translational repression but can also positively regulate gene transcription by targeting promoter elements (9).

MiR-146a is a dominant negative regulator of innate immune responses (10), but it is also has an effect on cell death/survival and differentiation (11). Stimulation with lipopolysaccharide (LPS) induced NF-κB dependent expression of miR-146a in THP1 cells (12). IL-1 receptor associated kinase (IRAK1) and TNF receptor-associated factor 6 (TRAF6) were identified as target genes for miR-146a mediated repression. These findings led to the well-acknowledged theory that miR-146a participates in a negative feedback loop, where the miR-146a expression is upregulated by, and acts as a repressor of signaling proteins of the innate immune response to avoid an overactive inflammatory response (12). This loop is initiated when LPS is recognized by the PRR toll like receptor 4 (TLR4). TLR4 signals through MyD88 (myeloid differentiation primary response protein 88), which recruits various signaling proteins including IRAK1 and TRAF6 that in turn activate the IKK complex (13). The activated IKK complex phosphorylates the inhibitory IκB, which is then degraded releasing the transcription factor NF-κB. NF-κB translocates to the nucleus, where it induces transcription of miR-146a along with various inflammatory cytokines (14). After processing, the RISC loaded miR-146a will then act as a negative regulator of IRAK1 and TRAF6, thus reducing NF-κB activation and completing the feedback loop. Further studies have shown that miR-146a expression is also induced by bacterial lipoprotein (BLP) and peptidoglycan acting through TLR2 and flagellin acting through TLR5 (12, 15).

We have previously shown that miR-146a is also upregulated in response to cuprizone (CPZ) exposure in mice, and that miR-146a deficiency protected against CPZ induced demyelination (16). Infusion of exogenous miR-146a mimics promoted remyelination, decreased M1-like phenotype and increased M2-like phenotype of microglia in the CPZ model (17). MiR-146a deficiency is also associated with a more severe EAE phenotype (18).

Oral administration of cuprizone (CPZ) in mice is an experimental model for de- and remyelination in MS (19). CPZ exposure is accompanied by microglial activation already 1 week after CPZ exposure, before demyelination can be detected (20). The number of activated microglia then increases during CPZ exposure and peaks at 4 weeks (7). Activated microglia within the demyelinating areas of CPZ lesions express various cytokines, chemokines and growth factors (21, 22), and inefficient clearance of myelin debris by microglia impairs remyelinating processes in the CPZ model (23). During the acute phase of remyelination in the CPZ model, a large number of genes are transcribed that suggest microglia activation, and among them are orthologs of differentially expressed genes also in MS lesions (24).

In this study, we investigated the cellular source of miR-146a in the non-injured mouse CNS, and found the highest level in postnatal microglia. Therefore, we examined the role of miR-146a in microglia activation *in vitro*, and in response to experimental de- and remyelination in the CPZ mouse model *in vivo*. We also isolated microglia from WT and miR-146a KO mice exposed to CPZ and investigated regulated proteins by label free quantitative proteomics. Finally, since miR-146 have been previously detected among differentially expressed miRNAs in MS lesions (25), we examined, which lesion types express miR-146a and its target genes in the white matter of MS brains.

## Materials and Methods

### Mice

The miR-146a KO mouse strain applied in this study was generated on a C57Bl/6 background in Dr. David Baltimore's laboratory, California Institute of Technology (26). We acquired the mice from the Jackson Laboratory (ME, USA). Female C57BL/6 WT mice were obtained from Taconic Ltd. (Ry, Denmark). All animal experiments were conducted in accordance with guidelines and protocols approved by the Danish Animal Health Care Committee (approval NO: 2014-15-00369). Mice younger than 7 days old were used for magnetic beads cell sorting (MACS) or sorting by the “shake off” method, and female mice aged 7–8 weeks were included in the CPZ experiments. At this age, miR-146a KO mice do not display any autoimmune or inflammatory phenotype (26).

### Cuprizone Induced de- and Remyelination

In order to induce demyelination in mice, powdered standard chow was mixed with 0.4% cuprizone (Sigma Aldrich, MO, USA), and delivered to the mice via the diet as previously described (16). CPZ was administrated for 4 weeks (4 weeks demyelination: 4wD), 4 weeks followed by 2 days of regular feeding (acute remyelination: 2dR) or 4 weeks followed by 2 weeks of regular feeding (full remyelination, 2wR). Control mice were kept on a normal chow diet.

### Microglia Isolation

#### *Ex vivo* Magnetic Beads Cell Sorting (MACS)

Brain dissociated to obtain single cell suspensions was performed by using MACS dissociation kits (Miltenyi Biotec, Lund, Sweeden,). Cells were labeled with CD140a Microbead Kit (oligodendrocyte precursor cells, OPCs), CD11b Microbead Kit (microglia), ACSA-2 Microbead Kit (astrocytes), O4 Microbead Kits (premature oligodedrocytes) or Neuron Isolation Kit. Labeled cells were loaded on a LS Column for positive selection (CD140a, CD11b, ACSA-2, and O4 Microbead Kits) or an LD column for negative selection (Neuron Isolation Kit), and placed in the magnetic field of a SuperMACS^TM^ II separator. The purity of the obtained cell fractions was further analyzed on a FACSCalibur^TM^ flow cytometer using FSCSDiva^TM^ software version 6.1.2 (BD Biosciences), and the obtained cell fractions were on average 74–98% pure. Microglia sorted by CD11b for *in vitro* stimulation assays were on average 90% pure, when characterized as CD11b^+^/CD45^low^ cells on the flow cytometer.

#### *In vitro* Sorting by Shaking Method

For investigation of phagocytosis and migration microglia were isolated by the “shake off” method (27). In short, CNS cells were seeded in a T75 flask after enzymatic dissociation and allowed to proliferate for 10 days. Then the flasks were shaken for 6 h at 200 rpm causing microglia to detach from the astrocyte monolayer. Microglia sorted this way were on average 95% pure when characterized as CD11b^+^/CD45^low^ on the flow cytometer.

### Antibodies and Fluorescence Activated Cell Sorting (FACS)

Cells were stained with PE-anti mouse CD45 (Biolegend, Copenhagen, Denmark) and PerCP-Cy5.5-anti-mouse CD11b (for sorting of whole microglia population) or PerCP-Cy5.5-anti-mouse CD11b, biotinylated-anti-mouse CD11c (BD pharmingen) and PE-anti mouse CD45 (for analysis of CD11c^+^ microglia) as previously described (8), and sorted by FACSAria^TM^ III cell sorter (BD Biosciences, Albortslund Denmark).

### Filter-Aided Sample Preparation for Proteomics of FACS Sorted Microglia

In total, between 180 and 1300 k cells were used for filter-aided sample preparation (FASP) for mass spectrometry, essentially as previously described (28), and cells for each sample was obtained from 3 to 5 brains. In brief, cells were lysed in 0.5% sodium deoxycholate in 50 mM triethylammonium bicarbonate (pH 8.6), denatured by heating to 95°C for 10 min, and ultrasonicated for 10 min. The protein concentration was determined by protein A280 (Thermo Nanodrop). A total of 100 μg from each sample was then reduced and alkylated using 10 mM Tris(2-carboxyethyl)phosphine hydrochloride, and 25 mM chloroacetamide for 30 min at room temperature. The samples were then incubated at 37°C with 1:100 (w/w) trypsin (mass spectrometry grade) per sample overnight. The peptides were extracted by solvent phase transfer, and stored at −80°C until LC-MS/MS.

### Mass Spectrometry Analysis

The individual samples (5 μg) were separated and sequenced using a ThermoSci QExactive High-Field orbitrap mass spectrometer in biological (*n* = 2–4 in each treatment, type) and technical duplicates (*n* = 2). In brief, a UPLC-nanoESI MS/MS setup with a Dionex RSLC nanopump (Dionex/Thermo Scientific, Waltham, USA) connected to Q-Exactive High-Field mass spectrometer (Thermo Scientific, Waltham, USA). The peptide material was loaded onto a C18 reversed phase column (Dionex Acclaim PepMap RSLC C18, 2 μm, 100 Å, 75 μm x 2 cm) and separated using a C18 reversed phase column (Dionex Acclaim PepMap RSLC C18, 2 μm, 75 Å, 75 μm ×75 cm) kept at 60°C.

A full MS scan in the mass range of m/z 375 to 1,500 was acquired at a resolution of 120,000. In each cycle, the mass spectrometer would trigger up to 10 MS/MS acquisitions of eluting ions based on highest signal intensity for fragmentation. The MS/MS scans were acquired with a dynamic mass range at a resolution of 15,000. The precursor ions were isolated using a quadrupole isolation window of m/z 2.0 and fragmented using higher-energy collision (HCD) with normalized collision energy of 27 and fixed first m/z of 110. Fragmented ions were dynamically added to an exclusion list for 15 s.

### Raw File Analysis of Mass Spectrometry Data

All MS scans were searched against the mouse isoform proteome from UniProt (date stamp 17MAR2017; 70.939 isoforms), which included indexed retention time standard (iRT standard) using MaxQuant/Andromeda v1.5.8.3.

The results were processed using Perseus v 1.5.8.3. Initially, all reverse database hits and proteins tagged as contaminants were removed before further analysis, and the LFQ intensity data was log2-transformed. The protein abundances were investigated duplicate-wise and raw data investigated by Persons correlation (>0.85). Principle components analysis was performed by imputing missing values from a normal distribution (width 0.3 and down shift 1.8). All protein groups with <50% values in each grouping (treatment, type) and unique peptides <1 were removed.

Proteins with statistically significant abundance change between exposure (CPZ vs. Ctrl) or strain groups (WT vs. KO) were identified for each group, and treatment using a permutation based FDR corrected *p-*value based two-sample students *t-*tests between treatment groups (FDR = 0.05 or 0.15, S0 = 0.1 and 250 randomizations).

Data was imported into R (v 3.6.1) using PerseusR (v 0.3.4) in Rstudio (v 1.2.5001) (29); https://www.R-project.org/ and http://www.rstudio.com/). The MixOmics package (v 6.8.5) was then used to perform a sparse Partial Least Squares-Discriminant Analysis (sPLS-DA), a supervised model that can be used to identify the most discriminative variables for classifying mice according to their respective group (CTL-KO, CTL-WT, CPZ-KO & CPZ-WT) (30). The performance of the sPLS-DA model was assessed using the MixOmics perf and tune.splsda functions with leave-one-out cross validation. The lowest classification error rate was achieved using 3 components with 7, 10, and 50 variables on component 1, 2, and 3, respectively. The selected variables on each component were used to create a total of 3 clustered image maps based on hierarchical clustering using the MixOmics cim function. STRING (v 11: https://string-db.org) was used to analyze known functional protein interactions of the selected variables from the sPLS-DA (31). Finally, the MixOmics pca function was used to perform a principal component analysis.

The mass spectrometry proteomics data have been deposited to the ProteomeXchange Consortium via the PRIDE repository with the dataset identifier PXD015939 (32, 33). The raw data supporting the conclusions of this manuscript will be made available by the authors, without undue reservation, to any qualified researcher.

### STRING Pathway Enrichment Analysis

Gene names of encoded proteins identified in the proteomics analysis were uploaded into the online STRING database (version 11: https://string-db.org). From this database we extracted a list of enriched KEGG pathways and compared the lists of enriched pathways between treatment groups by simple Venn diagram analysis (VENNY 2.1 http://bioinfogp.cnb.csic.es/tools/venny/).

### RNA Extractions and Quantitative PCR

Whole RNA was extracted using a miRNeasy micro kit (Qiagen, Denmark, Copenhagen). TaqMan chemistry primers were acquired from Life Technologies (miR-146a ID: 000468, sno135 ID: 001230, U6: 001973, Thermo Scientific, CA, USA) and RNA was transcribed using a TaqMan microRNA reverse transcription kit (Life Technologies, Thermo Scientific). qPCR was performed on Bio-Rad CFX Connect^TM^ Real-Time System (software Bio-Rad CFX Manager 3.1). Expression levels were reported relative to geometric mean of snoRNA135 and U6snRNA. TNF expression was analyzed as previously described (34).

### *In vitro* Stimulation of Microglia Cells

Microglia (20.000 to 200.000) were seeded in each well of poly-l-lysine coated standard plates in DMEM media (Sigma Aldrich). After 2 h, cell culture media was changed and new media containing LPS (10 ng/ml) or myelin (0.2 ng/ml or 50 ng/ml) was added. Cell culture media was collected, or cells were lyzed directly in the wells 24 h after LPS stimulation, or 48 h after myelin stimulation.

### Meso Scale Discovery (MSD) Multiplex Analysis

Cytokine levels in cell culture media were measured by the Meso Scale Discovery (MSD, USA) electrochemiluminescence proinflammatory mouse V-Plex Plus Kit and a MULTI-SPOT 4 spot cytokine costume plate (CCL2, CCL3, VEGF and MMP9). A SECTOR Imager 6,000 (Meso Scale Discovery) Plate Reader was used for analysis and data were analyzed using the MSD Discovery Workbench software according to the manufacturer's instructions.

### Phagocytosis Assay

Phagocytotic activity was examined by measuring fluorescence of internalized latex beads by flow cytometer (35) (Supplementary Figure 1). Microglia were stimulated with LPS or myelin for 24 h. Fluorescent latex beads were added for 40 min at 37°C (samples) or 4°C (controls). Cytochalasin D (5 μg/ml), was added as a negative control. Incubation was stopped by placing the cells on ice. Cells were detached from the plate by adding 0, 2% Trypsin-EDTA to the cells, and analyzed by flow cytometry.

### Random Migration Assay

Microglia were seeded at a density of 13.000 cells/well in a PDL coated 96-well ImageLock Plate (Essen Bioscience) and stained with CellTracker Red CMTPX Fluorescent Probes (Life Technologies). Cells were stimulated with LPS or myelin and placed in the IncuCyte Zoom live cell imagining system (Essen BioScience, USA). Pictures were taken every 20 min for 24 h. Single cell motility was quantified using the Fiji plugin TrackMate for semi-automated particle tracking (36). In TrackMate, the Laplacian of Gaussian (LoG) detector with estimate spot diameter of 21 μm and a threshold of 0.5 was used to detect individual cells. The simple linear assignment problem (LAP) tracker with a linking maximum distance of 80 μm, a gap-closing max distance of 80 μm and a gap closing max distance of 2 was used to track cell migration through the time course movies.

### Lesion Classification and RNA Extraction From Specific Histological Brain Areas of Patients With MS

Examination of mir-146a expression in human progressive white matter (WM) MS brain tissue was done on 8 normal-appearing white matter (NAWM) areas and 8 active lesions (AL) (*n* = 8). WM areas (*n* = 8) of non-neurological diseased cases were used as controls. The RNA was collected from our previously study (37), and the mir-RNA level of mir-146a and the reference genes (sno135, U6) were measured as described above using the TaqMan microRNA reverse transcription and TaqMan chemistry primers.

For finding validated target genes of miR-146a, we used the miRTarBase (38, 39). To identify the target genes significantly expressed in different stages of lesion evolution in the brain white matter of MS patients we used the MS-Atlas (www.msatlas.dk).

### Statistics

All statistical tests were performed, and graphs were conducted using Prism 5 software (GraphPath, USA, CA). One significant outlier according to the Grubbs' test of extreme studentized deviates was removed from the cellular source dataset, and one from the 48-h time point analysis of CCL3 after 0.2 ng/ml myelin stimulation. Cellular source analysis and analysis of miR-146a levels in response to LPS stimulation, and analysis of miR-146a expression in microdissected MS brain lesions were performed using a parametric one-way AVOVA test. Migration, phagocytosis, MSD data analysis, miR-146a expression levels in response to myelin stimulation, CD11c^+^ cells analysis and percentage of CD11b^+^/CD45^high^ cells were analyzed using a two-way ANOVA test, followed by appropriate *post hoc* tests, and the level of microglia cells in naïve adult brains was analyzed using a parametric student *t*-test. Data are presented as mean ± SD. Statistics for proteomics analysis is described in detail in the section “Raw File Analysis of mass spectrometry data.”

## Results

### Microglia Express High Levels of miR-146a Compared to Other Cell Types in the Brain

**Figure 1:**
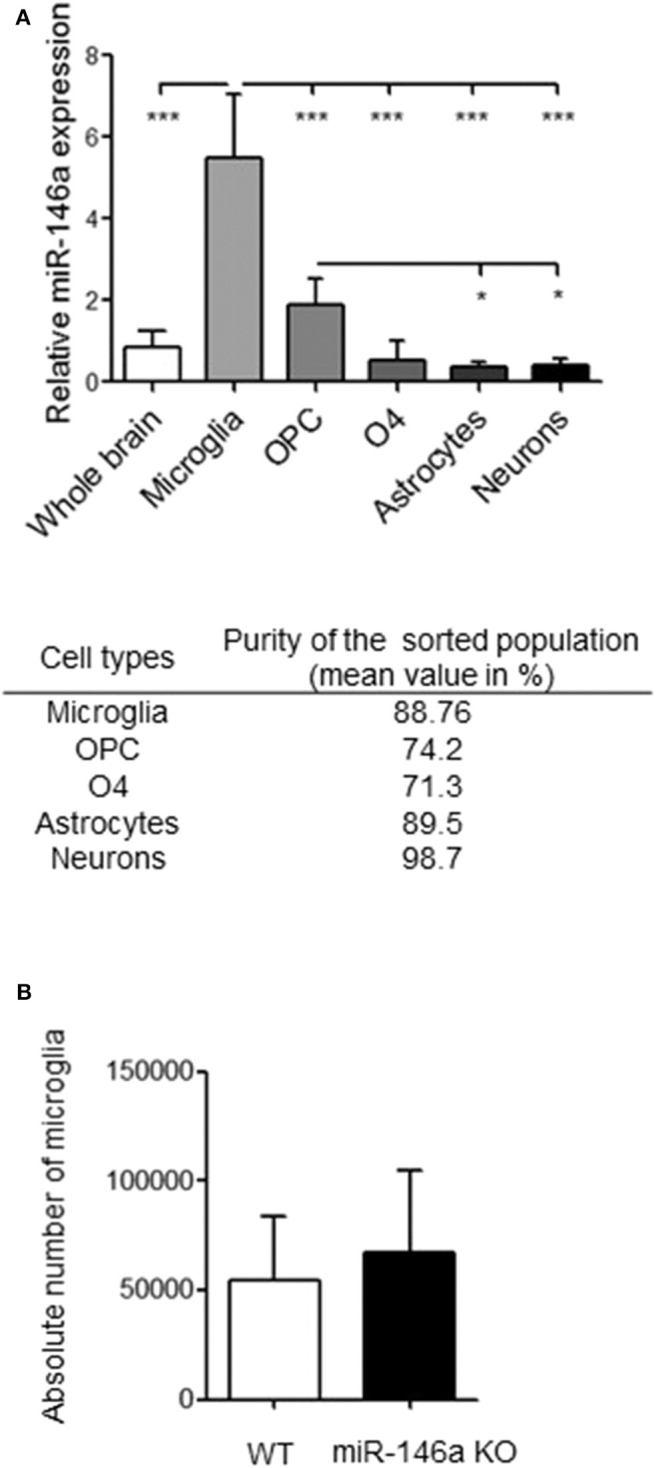
Expression of miR-146a in brain resident cells and microglia in miR-146a KO mice. **(A)** Using MACS, microglia were sorted by anti-CD11b, OPCs by anti-PDGFRα, immature oligodendrocytes by anti-O4, astrocytes by anti-ACSA-2, and neurons by negative selection. Purity of each population is indicated. Relative expression of miR-146a was determined by qPCR and expression levels are presented relative to geometric mean of U6snRNA and sno135 levels. Each sample is a pool of 3–5 brains. Statistics: *p < 0.05, ***p < 0.001, *n* = 5–8 in each group, one-way ANOVA followed by Tukey's multiple comparisons test, mean ± SD. **(B)** The number of microglia was analyzed by flow cytometry, and microglia were defined as CD11b^+^/CD45^low^ cells. *n* = 6–7 in each group, parametric students t-test, mean ±SD.

To identify the main cellular source of miR-146a in the brain by qPCR, microglia (CD11b^+^ cells), OPCs (CD140a^+^ cells), premature oligodendrocytes (O4^+^ cells), astrocytes (ACSA-2^+^ cells) and neurons (negative selection) were isolated from the brains of postnatal mice by MACS. Microglia expressed miR-146a at the highest level followed by OPCs, that expressed miR-146a at higher level than astrocytes and neurons (Figure 1A). We also analyzed the level of miR-146a in isolated adult microglia compared to the entire CNS cell fractions without microglia: the expression of miR-146a was 7.6 times higher in the microglia fraction (Figure 1A).

Since miR-146a was expressed at the highest level in microglia, we next examined if the absence of miR-146a alters microglia functions.

### MiR-146a Deficient Mice Have Normal Levels of Microglia in the Brain

First, we investigated if the absence of miR-146a affects the number of microglia in the healthy mouse brain. Using flow cytometry, we found that adult naive miR-146a KO mice and WT mice had similar number of CD11b^+^CD45^low^ microglia (Figure 1B).

### Cytokine/Chemokine Production of LPS-Activated Microglia Is Increased in the Absence of miR-146a

**Figure 2:**
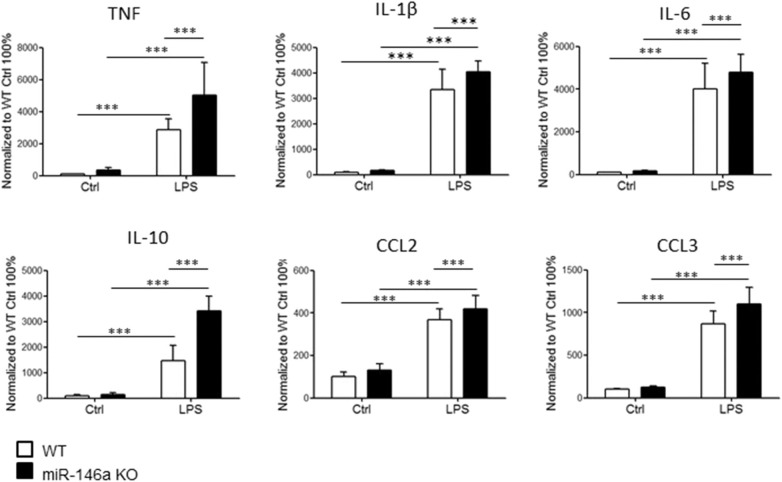
Expression of cytokines and chemokines by miR-14a KO and WT microglia stimulated with LPS. Microglia were sorted by CD11b microbeads from postnatal miR146a-KO mice and WT mice and stimulated with LPS (10 ng/ml) for 24 h *in vitro*. The level of cytokines and chemokines was measured by MSD multiplex analysis. Statistics: ***p < 0.001, *n* = 11–21 in each group, two-way ANOVA followed by Bonferroni posttest, mean ± SD.

Next, we investigated cytokine and chemokine production by LPS-stimulated miR-146a KO and WT microglia. Microglia were stimulated for 24 h *in vitro*, and the levels of cytokines and chemokines in the cell culture media were examined by MSD multiplex analysis. We found increased levels of TNF, IL-6, IL-1β, IL-10, CCL2, and CCL3 in in miR-146a KO compared to WT microglia cultures (Figure 2).

### MiR-146a Deficient Microglia Response to Myelin *in vitro*

**Figure 3:**
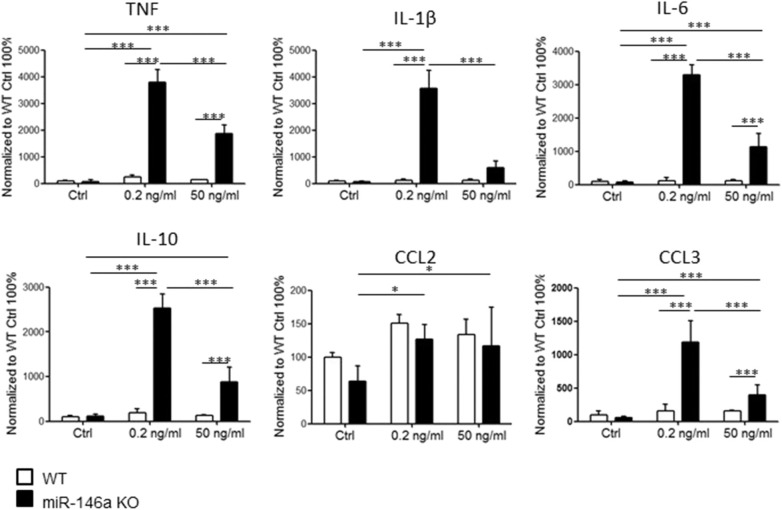
Expression of cytokines and chemokines by miR-14a KO and WT microglia stimulated with myelin. Microglia were sorted by CD11b microbeads from postnatal miR146a-KO mice and WT mice and stimulated with 0.2 ng/ml or 50 ng/ml myelin for 48 h *in vitro*. The level of cytokines and chemokines in the cell culture media was measured by MSD multiplex analyses. Statistics: *p < 0.05, ***p < 0.001, *n* = 3–4 in each group, Two-way ANOVA followed by Bonferroni posttest, mean ± SD.

Knowing that microglia cells are highly activated by demyelination pathologies *in vivo* (7, 21, 22), we next investigated the response to myelin stimulation *in vitro*. Postnatal microglia were sorted from WT mice and miR-146a KO mice by MACS and stimulated with either 0.2 ng/ml or 50 ng/ml myelin for 48 h. We found that miR-146a KO microglia cells were strongly activated by myelin stimulation, and secreted IL-1β, TNF, IL-6, IL-10, CCL3, and CCL2 (Figure 3). None of the cytokines or chemokines were significantly changed in response to myelin stimulation in WT microglia; however, we observed a non-significant tendency of elevation for all cytokines and chemokines.

### Phagocytosis Is Reduced in Microglia in the Absence of miR-146a

**Figure 4:**
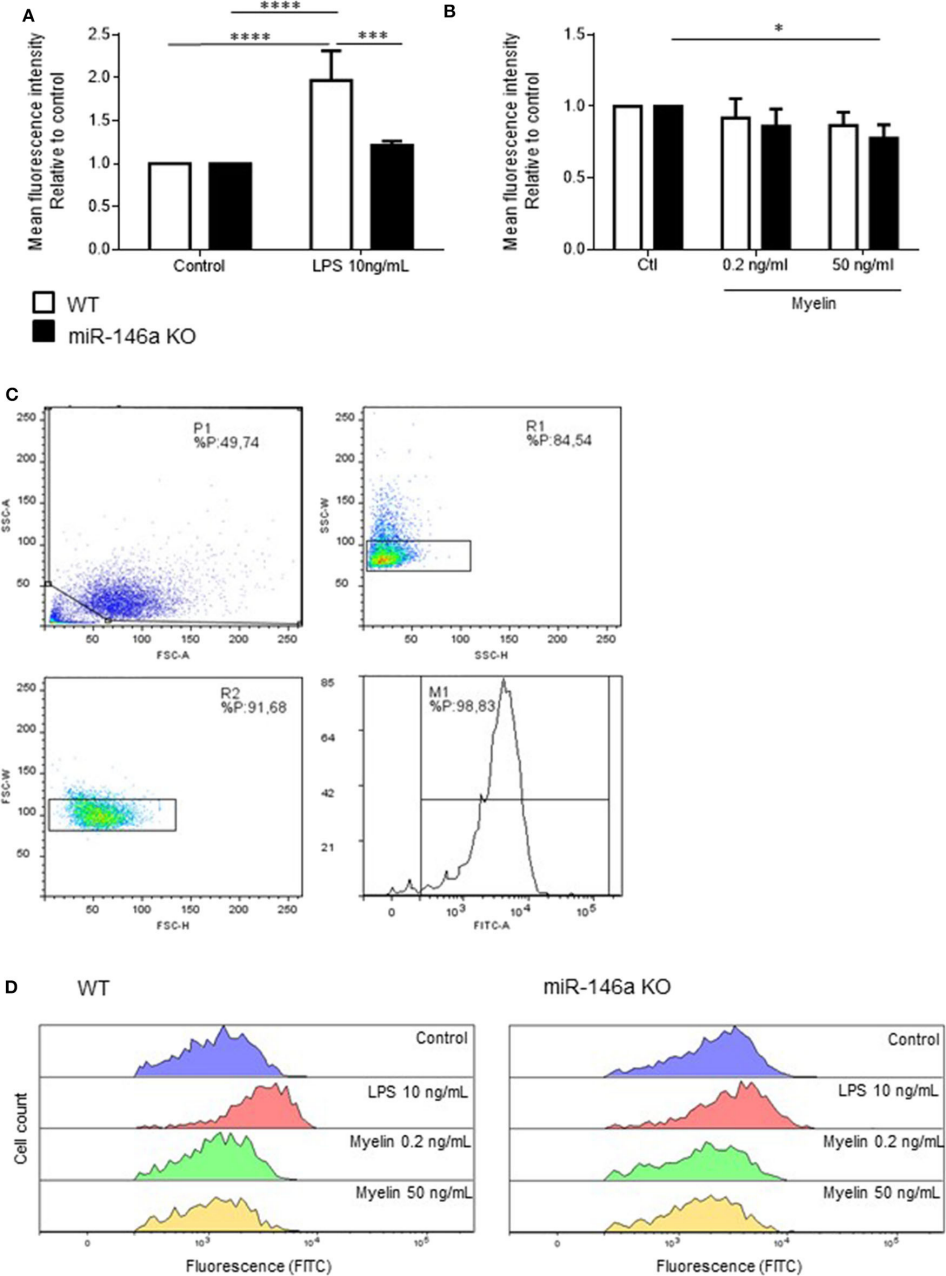
Phagocytosis by miR-146a KO and WT microglia stimulated with LPS and myelin. Microglia were treated with LPS **(A)** or myelin **(B)** for 24 h before the addition of fluorescent latex beads. Cells were allowed to ingest the beads for 40 min before the analysis made on a flow cytometer. Phagocytosis was quantified as mean fluorescence intensity and displayed relative to WT control. **(C)** Gating strategy: Initial gating to exclude cell debris and dead cells (upper left panel); doublet discrimination based on side scatter height and width (upper right panel); doublet discrimination based on forward scatter height and width (lower left panel); separation of labeled and unlabeled cells (lower right panel). **(D)** Example of fluorescein intensity of phagocytosed beads after stimulation with different concentration of LPS and myelin. Statistics: *p < 0.05, ***p < 0.01, ****p < 0.0001, n = 3–4 in each group, Two-way ANOVA followed by Tukey's posttest, mean ± SD.

Next, we investigated how *in vitro* stimulation with LPS, and myelin affected the phagocytotic ability of microglia (Figure 4). Postnatal WT and miR-146a KO microglia were stimulated with either 10 ng/ml LPS or 0.2 ng/ml and 50 ng/ml myelin for 24 h, and fluorescent latex beads were added to the activated microglia. We found that activation of microglia with LPS increased phagocytosis by WT microglia but not miR-146a KO microglia (Figure 4A). Activation with myelin did not increase phagocytosis, on the contrary, stimulation with 50 ng/ml myelin significantly decreased phagocytosis by miR-146a KO microglia (Figure 4B).

### Stimulation With LPS and Myelin Induce Random Migration in WT but Not in KO Microglia

**Figure 5:**
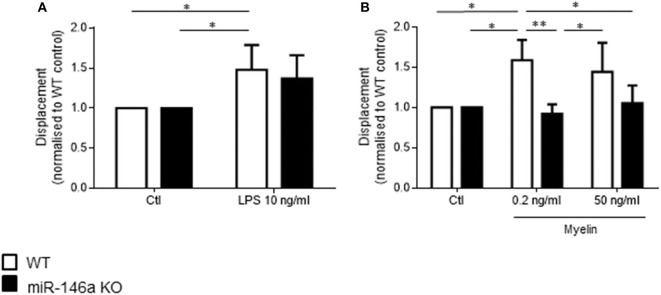
Random single cell migration by miR-146a KO and WT microglia stimulated with LPS and myelin. Single cell track displacement of microglia stimulated with LPS **(A)** or myelin **(B)** for 24 h was quantified by analysis with TrackMate. Statistics: *p < 0.05, **p < 0.01, *n* = 3 in each group, Two-way ANOVA followed by Sidak's posttest, mean ± SD.

We then investigated how *in vitro* stimulation with LPS and myelin affected random single cell microglia migration (Figure 5). Postnatal microglia were stimulated with 10 ng/ml LPS, 0.2 ng/ml myelin, or 50 ng/ml myelin for 24 h, and random migration of single cells was examined during this time by TrackMate. Activation of microglia with LPS and myelin increased random migration of WT microglia but not miR-146a KO microglia.

### Reduced *in vivo* Generation of CD11c^+^ Microglia in miR-146a KO Mice

**Figure 6:**
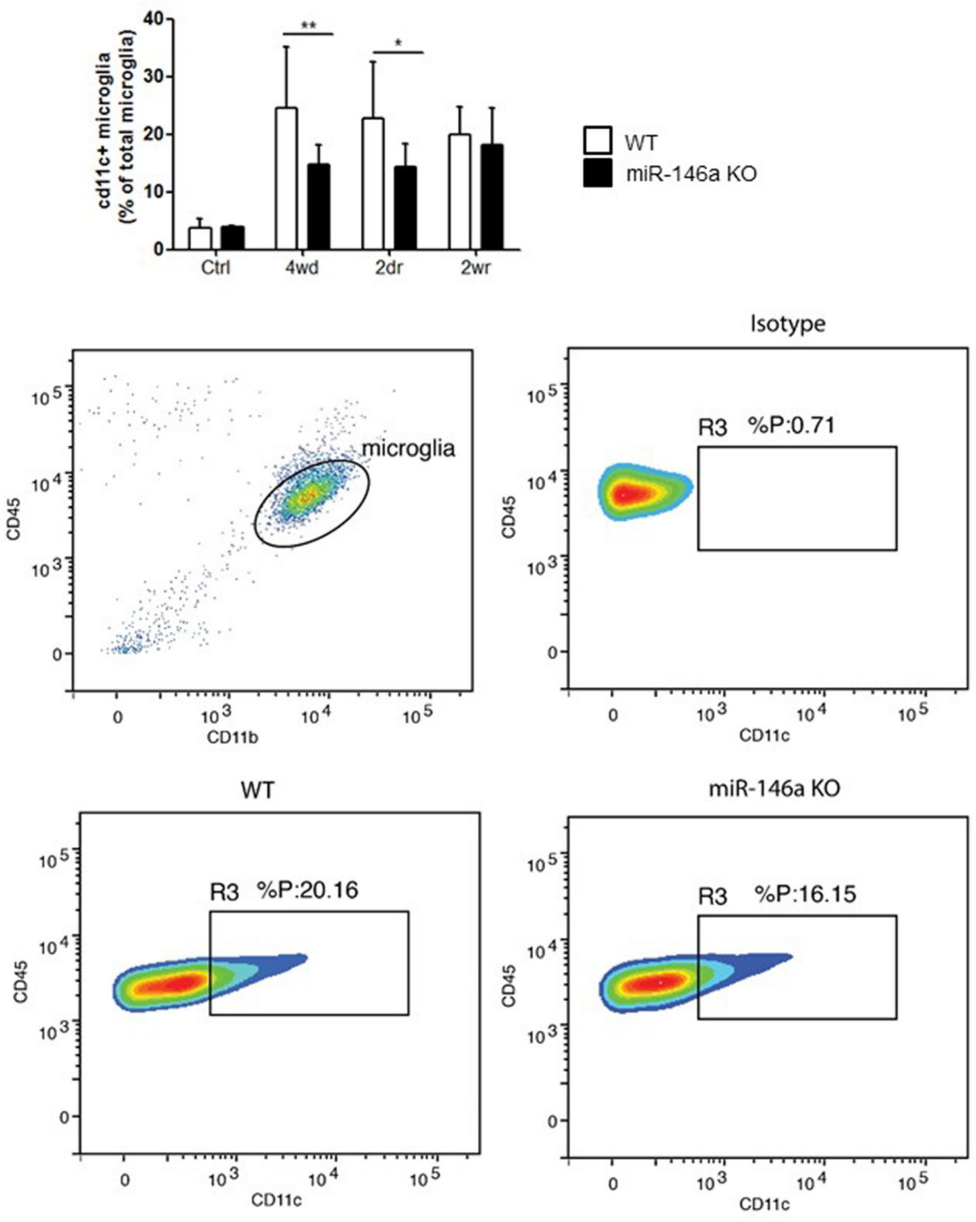
Upregulation of CD11c^+^ microglia in response to CPZ treatment is decreased in miR-146a KO mice. The level of CD11c^+^ microglia was measured in the brain of mice exposed to CPZ and compared between miR-146a KO and WT mice. This was made using flow cytometry. *p < 0.05, **p < 0.01, n = 4–12, Two-way ANOVA followed by Bonferroni *post hoc* test, mean ± SD. Ctrl, unmanipulated controls; 4wd, 4 weeks demyelination; 2dr, 2 days (acute) remyelination; 2wr, 2 weeks (full) remyelination.

In order to investigate the role of miR-146a in microglia activation in response to demyelination *in vivo*, we used the CPZ mouse model (7, 21, 22). We examined the number of CD11c^+^ microglia that is effective in antigen presentation, but poorly express proinflammatory cytokines (7, 8, 40). We found that the percentage of CD11c^+^ microglia increased in response to CPZ exposure in the WT mice, but the increase was lower in the miR-146a KO mice compared to WT mice during demyelination and acute remyelination (Figure 6).

### Proteomics Analysis of WT and miR-146a KO Microglia Isolated During CPZ-Induced Demyelination

**Figure 7:**
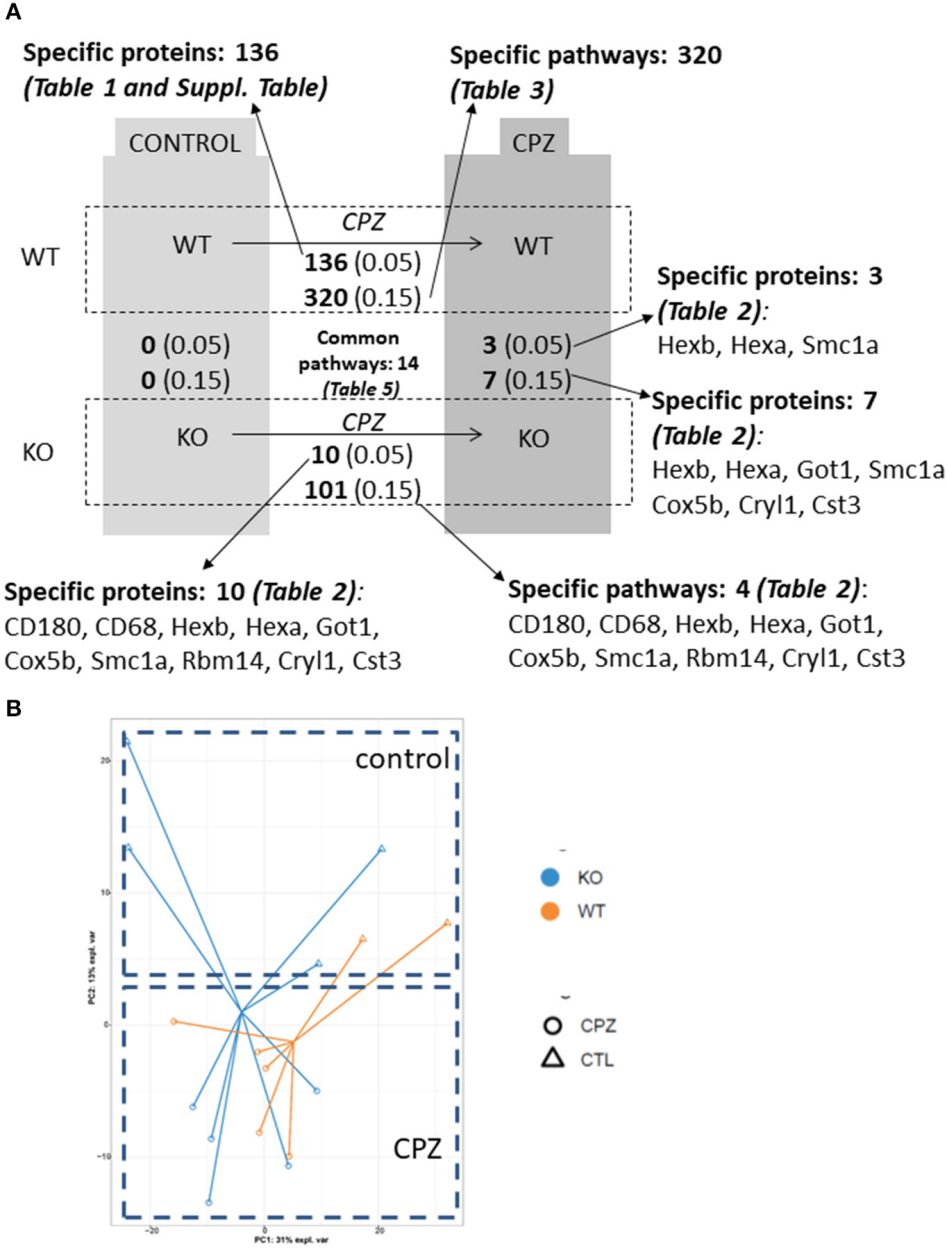
Quantitative proteome analysis of microglia proteome isolated from wild-type and miR-146 KO mice during cuprizone-induced demyelination. **(A)** Summary of the quantitative analysis based on multiple-hypothesis testing and relaxed statistical analysis indicating the number of specific microglia proteins and pathways that were uniquely regulated among 4 conditions: wild-type control mice, wild-type mice after 4-week cuprizone (CPZ) treatment, KO control, and KO mice after 4-week CPZ treatment. The number of common pathways is also shown. The corresponding proteins are listed in different tables that are also indicated in the figure. **(B)** Principle component analysis of isolated microglia proteomes shows separation of the 4 conditions: proteome of microglia isolated from WT and KO mice without cuprizone (CPZ) treatment (control, CTL) and after 4-week CPZ (demyelination).

Next, we determined the proteome of both resting microglia and *in vivo* activated microglia during CPZ-induced demyelination in adult WT and miR-146a KO mice (Figure 7A). Microglia cells were sorted as CD11b^+^, CD45^low^ cells from the brains of naïve (Ctrl) and CPZ-exposed WT and miR-146a KO mice, and the proteome was analyzed by mass spectrometry (UPLC-nanoESI MS/MS). We next investigated the overall similarities between the 32 LC-MS runs by an unsupervised PCA on the complete data set after merging the technical duplicates. The proteome of the resting microglia was not different between KO and WT microglia. However, the PCA analysis indicated a major difference between the CTL and CPZ groups (Figures 7A,B). We identified 1.549 unique proteins hereof 1,065 quantifiable proteins: 136 proteins were significantly changed in microglia sorted from WT mice in response to CPZ exposure (FDR <0.05). Out of these, 38 were upregulated whereas 98 were downregulated (Figure 7A, Table 1, Supplementary Table 1). In activated microglia sorted from the miR-146a KO mice during demyelination, we only identified 10 differentially regulated proteins (*p* < 0.05 FDR) out of which 6 were upregulated and 4 were downregulated (Figure 7A, Table 2). Out of these 10 proteins, 3 proteins (HEXA, HEXB, and SMC1A) were also differentially regulated in WT microglia (Tables 1, 2). When using a less stringent FDR threshold value of 0.15 for WT microglia, we could find 3 additional proteins out of the 10 proteins in the KO dataset (CD180, CD68, and RBM14) among the differentially regulated proteins in the WT mice, suggesting that the remaining 4 proteins out of the 10 proteins (GOT1, COX5B, CRYL1, and CST3) were dysregulated specifically in the miR-146a KO microglia in response to CPZ exposure.

**Table 1:** Top 25 dysregulated proteins in WT microglia in response to CPZ exposure *in vivo*.

**Protein**		**Change**	***P-*value**
**Top 25 upregulated proteins in WT microglia exposed to CPZ** ***in vivo***			
Alpha-2-macroglobulin-P	A2mp	3.29	0.0024
Apolipoprotein A-I	Apoa1	3.24	0.0046
Inter-alpha-trypsin inhibitor heavy chain H2	Itih2	3.11	0.0088
Apolipoprotein E	Apoe	2.20	0.027
Unconventional myosin-IXb	Myo9b	2.10	0.029
Thymosin beta-4;Hematopoietic system regulatory peptide	Tmsb4x	1.89	0.012
Beta-hexosaminidase subunit alpha	Hexa	1.58	0.0048
Apoptosis-associated speck-like protein containing a CARD	Pycard	1.37	0.031
40S ribosomal protein S10	Rps10	1.36	0.0074
Phosphoserine aminotransferase	Psat1	1.35	0.027
Cystatin-B	Cstb	1.32	0.0069
Cofilin-1	Cfl1	1.28	0.0017
H-2 class I histocompatibility antigen	H2-D1;H2-Q7;H2-Q6;H2-Q9;H2-Q8	1.26	0.023
Beta-hexosaminidase subunit beta	Hexb	1.14	0.013
Crk-like protein	Crkl	1.14	0.0028
Annexin A5	Anxa5	1.11	0.0015
V-type proton ATPase subunit F	Atp6v1f	1.01	0.0066
Thioredoxin	Txn	1.01	0.00017
Macrophage-capping protein	Capg	0.98	0.016
V-type proton ATPase subunit G 1	Atp6v1g1	0.96	0.00063
Macrophage migration inhibitory factor	Mif	0.94	0.019
Superoxide dismutase [Cu-Zn]	Sod1	0.89	0.0014
Elongation factor 1-beta	Eef1b2;Eef1b	0.86	0.0042
Poly [ADP-ribose] polymerase 1	Parp1	−3.55	0.021
Pre-mRNA-processing-splicing factor 8	Prpf8	−3.22	0.017
Guanine nucleotide-binding protein G(I)/G(S)/G(T) subunit beta-2	Gnb2	−2.57	0.00058
Centromere protein V	Cenpv	−2.55	0.026
Structural maintenance of chromosomes protein 1A	Smc1a	−2.54	0.022
Importin subunit alpha-4;Importin subunit alpha-3	Kpna3;Kpna4	−2.52	0.0083
Host cell factor 1	Hcfc1	−2.40	0.00078
Sulfide:quinone oxidoreductase, mitochondrial	Sqrdl	−2.40	0.026
U5 small nuclear ribonucleoprotein 200 kDa helicase	Snrnp200	−2.26	0.013
Heterogeneous nuclear ribonucleoprotein H2	Hnrnph2	−2.19	0.0047
Lysophosphatidylcholine acyltransferase 2	Lpcat2	−2.19	0.022
Cleavage and polyadenylation specificity factor subunit 5	Nudt21	−2.19	0.000064
Interleukin enhancer-binding factor 2	Ilf2	−2.17	0.00038
Splicing factor 3B subunit 1	Sf3b1	−2.12	0.0039
P2Y purinoceptor 12	P2ry12	−2.09	0.019
Structural maintenance of chromosomes protein 3	Smc3	−2.07	0.019
THO complex subunit 2	Thoc2	−2.04	0.011
N-acylneuraminate cytidylyltransferase	Cmas	−2.03	0.010
Integrin alpha-M	Itgam	−2.03	0.0075
4-aminobutyrate aminotransferase, mitochondrial	Abat	−1.98	0.035
Prohibitin-2	Phb2	−1.98	0.019
Myelin expression factor 2	Myef2	−1.98	0.0018
SUN domain-containing protein 2	Sun2	−1.96	0.023
Band 4.1-like protein 2	Epb41l2	−1.95	0.0029
RuvB-like 2	Ruvbl2	−1.94	0.033

**Table 2:** Dysregulated proteins in miR-146 KO microglia in response to CPZ exposure *in vivo*.

**Protein**		**Change**	***P-*value**
CD180 antigen	Cd180	2.031	0.000055
Macrosialin	Cd68	2.028	0.0029
Beta-hexosaminidase subunit beta	Hexb	2.35	0.00021
Beta-hexosaminidase subunit alpha	Hexa	2.31	0.000013
**Aspartate aminotransferase, cytoplasmic**	**Got1**	**0.99**	**0.00060**
**Cytochrome c oxidase subunit 5B**,	**Cox5b**	**1.22**	**0.000097**
**mitochondrial**			
Structural maintenance of chromosomes	Smc1a	−2.25	0.0032
protein 1A			
RNA-binding protein 14	Rbm14	−1.68	0.0028
**Lambda-crystallin homolog**	**Cryl1**	–**1.53**	**0.0024**
**Cystatin-C**	**Cst3**	–**1.27**	**0.000084**

*Proteins in bold were dysregulated only in the miR-146a KO mice*.

**Figure 8:**
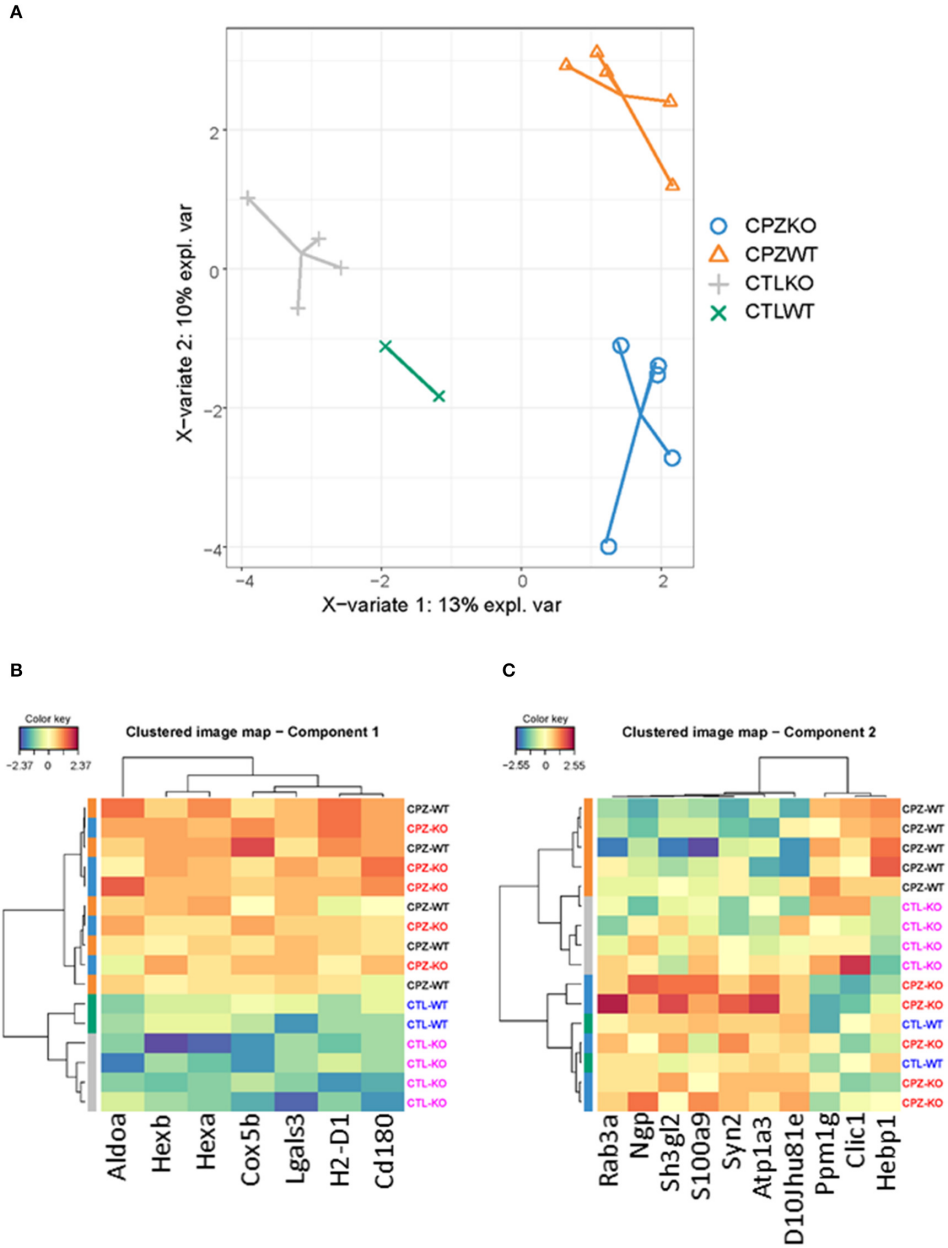
Supervised investigation of co-regulated microglia proteins isolated from wild-type and miR-146 KO mice during cuprizone-induced demyelination. **(A)** The supervised model, Partial Least Squares-Discriminant Analysis (sPLS-DA) was used to identify discriminative proteins in the proteome of microglia isolated from wild-type control mice, wild-type mice after 4-week cuprizone (CPZ) treatment, KO control, and KO mice after 4-week CPZ treatment. Component 1 (X axis) was able to separate microglia according to proteome with or without CPZ treatment, while component 2 separated wild-type and KO microglia after 4-week CPZ treatment (Y axis). **(B)** Hierarchical clustering of proteins in component 1. **(C)** Hierarchical clustering of proteins in component 2. See also Supplementary Table 2.

**Figure 9:**
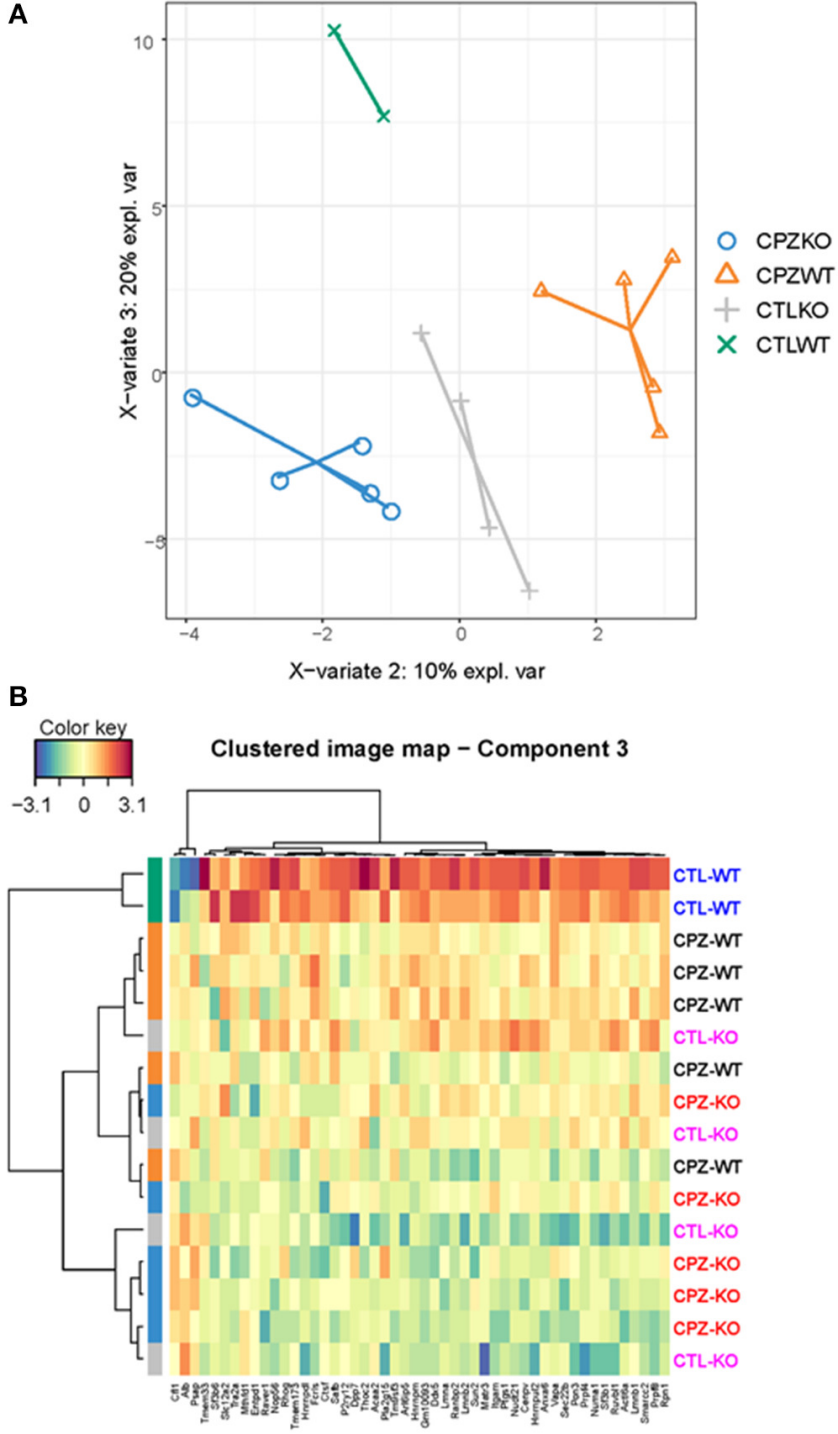
Supervised analysis of microglia proteome isolated from wild-type and miR-146 KO mice during cuprizone-induced demyelination. **(A)** The supervised model, Partial Least Squares-Discriminant Analysis (sPLS-DA) was used to identify discriminative proteins in the proteome of microglia isolated from wild-type control mice, wild-type mice after 4-week cuprizone (CPZ) treatment, KO control, and KO mice after 4-week CPZ treatment (*see Methods*). Component 3 (Y axis) separated control wild-type microglia from all the other groups by 50 proteins. **(B)** Hierarchical clustering of 50 regulated proteins in component 3. See also Supplementary Table 2.

To address discriminative features of proteins among the microglia proteomes of the wild-type and KO-mice without (control) or with CPZ treatment, we used the sparse Partial Least Squares-Discriminant Analysis (sPLS-DA). The lowest classification error rate was achieved using 3 components with 7, 10, and 50 variables on component 1, 2, and 3, respectively (Figures 8A, 9A). The selected variables on each component were used to create a total of 3 clustered image maps based on hierarchical clustering using the MixOmics cim function (Figures 8B,C, 9B).

sPLS-DA was able to discriminate CPZ treated mice from the control mice on component 1 (Figure 8A). The clustered image map that was made using the seven variables for driving the discrimination on component 1, showed a clear grouping of the mice according to treatment (Figure 8B). Further, the clustered image map showed that these proteins were upregulated in the CPZ-treated mice and downregulated in the control mice (Figure 8B). The STRING analysis of these proteins (Supplementary Table 2) showed significant interactions that is related to ganglioside catabolic and metabolic process, lipid storage, carbohydrate derivative metabolic process and lysosome organization (data not shown).

The sPLS-DA was also able to separate the CPZ-WT and CPZ-KO mice on component 2 (Figure 8A). The clustered image map showed a cluster of ten proteins that was downregulated in CPZ-WT and upregulated in CPZ-KO (Figure 8C). The STRING analysis of these proteins (Supplementary Table 2) showed significant interactions related to regulation of plasma membrane organization, synaptic vesicle recycling, vesicle-mediated transport in synapse and neurotransmitter secretion (data not shown).

Finally, the sPLS-DA was able to separate the control WT from all the other groups on component 3 (Figure 9A). The clustered image map showed a collection of 50 proteins that was upregulated in control WT (Figure 9B). The STRING analysis of these proteins (Supplementary Table 2) showed significant interactions related to mRNA metabolic process, mRNA processing, mRNA splicing via spliceosome and RNA splicing, but also biological processes related to negative regulation of amine transport, regulation of cellular protein localization and negative regulation of catecholamine secretion (data not shown).

### Pathway Analysis of Differentially Regulated Proteins in WT and miR-146a KO Microglia in Response to CPZ

Next, we aimed to identify pathways that characterized the activated miR-146a KO microglia response to CPZ exposure *in vivo*.

Because of the low number of dysregulated proteins in the miR-146a KO microglia by FDR <0.05 threshold, the pathway analyses were conducted using the less stringent FDR-value of 0.15. Pathway analyses of WT microglia and miR-146a KO microglia thus included 320 and 101 dysregulated proteins in response to CPZ-induced demyelination, respectively.

By using STRING pathway enrichment analysis, we determined a list of KEGG pathways that were significantly enhanced in the WT microglia, and another list of significantly enhanced pathways in the miR-146a KO microglia. The two lists were then compared to identify WT specific pathways (Table 3), miR-146a KO specific pathways (Table 4) and pathways changed in both WT and miR-146a KO microglia (Table 5). We identified 16 WT-specific pathways, which were not changed in the miR-146a KO microglia (Table 3), whereas we only identified 4 miR-146a KO-specific pathways (Table 4). Two of these 4 pathways contained only proteins which were also changed in the WT microglia (glycosphingolipid biosynthesis—globo series, and glycosphingolipid biosynthesis—ganglio series), and therefore the significant increase seen in these pathways, may be a statistical artifacts. The other two pathways, cysteine and methionine metabolism and cGMP-PKG signaling pathway contained some proteins only found in the miR-146a KO dataset (GOT1 and VDAC2). Although most proteins were changed in both datasets most pathways that were dysregulated in the miR-146a KO mice were also altered in the WT mice (Table 5).

**Table 3:** Dysregulated pathways specific to WT microglia in response to CPZ exposure *in vivo*.

**WT specific KEGG pathways**	**FDR**	**Changed pathway members**
Valine, leucine and isoleucine degradation	6.4e-06	Abat, Acaa2, Acat1, Aldh3a2, Aldh6a1, Dld, Ivd, Pccb
Proteasome	2.74e-05	Psma5, Psmb10, Psmb6, Psmb8, Psme1, Psme2, Psme3
Alzheimer s disease	0.00017	Apoe, Atp5a1, Atp5c1, Atp5j, Cox7a2, Ndufa4, Ndufv1, Sdha, Uqcrc1, Uqcrc2, Uqcrfs1
Antigen processing and presentation	0.00069	Canx, Ctsb, Ctsl, Hspa4, Psme1, Psme2, Psme3
mRNA surveillance pathway	0.0022	Dazap1, Nudt21, Ppp1ca, Ppp1cb, Ppp1cc, Rnmt, Wdr82
Glyoxylate and dicarboxylate metabolism	0.0039	Acat1, Aco2, Cat, Pccb
Amino sugar and nucleotide sugar metabolism	0.0049	Cmas, Cyb5r3, Hexa, Hexb, Hk1
beta-Alanine metabolism	0.0082	Abat, Aldh3a2, Aldh6a1, Cndp2
Oocyte meiosis	0.19	Ppp1ca, Ppp1cb, Ppp1cc, Smc1a, Smc3, Ywhab
Fatty acid degradation	0.031	Acaa2, Acat1, Aldh3a2, Eci1
Cardiac muscle contraction	0.22	Atp1b1, Cox7a2, Uqcrc1, Uqcrc2, Uqcrfs1
Non-alcoholic fatty liver disease (NAFLD)	0.23	Cox7a2, Ndufa4, Ndufv1, Sdha, Uqcrc1, Uqcrc2, Uqcrfs1
Protein export	0.25	Spcs1, Spcs2, Spcs3
Herpes simplex infection	0.28	C1qbp, C3, Gtf2i, Hcfc1, Ppp1ca, Ppp1cb, Ppp1cc, Srsf7
RNA transport	0.32	Kpnb1, Nup155, Nup210, Ranbp2, Thoc2, Thoc6, Tpr
Collecting duct acid secretion	0.32	Atp6v1e1, Atp6v1f, Atp6v1g1

**Table 4:** Dysregulated pathways specifc to miR-146a microglia in response to CPZ exposure *in vivo*.

**KO specific KEGG pathways**	**FDR**	**Changed protein members**
Cysteine and methionine metabolism	3.45e-05	Apip, Got1, Ldhb, Mdh1, Mdh2
cGMP-PKG signaling pathway	0.12	Atp1b1, Gtf2i, Ppp1ca, Vdac1, Vdac2
Glycosphingolipid biosynthesis—globo series	0.24	Hexa, Hexb
Glycosphingolipid biosynthesis—ganglio series	0.24	Hexa, Hexb

**Table 5:** Dysregulated pathways enriched in both WT and miR-146a KO microglia in response to CPZ exposure *in vivo*.

**Strain**	**KEGG pathway**	**FDR**	**Changed protein members**
WT	Spliceosome	1.61e−16	Cdc5l, Ddx42, Ddx5, Dhx15, Hnrnpc, Hnrnpm, Prpf19, Prpf4, Prpf8, Puf60, Sf3a1, Sf3a3, Sf3b1, Sf3b3, Sf3b6, Snrnp200, Snrnp40, Snrpd1, Snrpe, Srsf7, Thoc2, U2af2
KO		0.0070	Cdc5l, Lsm3, Sf3b1, Snrnp200, Srsf9
WT	Microbial metabolism in diverse environments	9.88e−09	Acaa2, Acat1, Aco2, Acyp1, Akr1a1, Aldh3a2, Aldoa, Cat, Dlat, Dld, Hk1, Idh2, Ldha, Pccb, Pdhb, Pgam1, Pon3
KO		4.42e−05	Aldoa, Echs1, Eno2, Ldhb, Mdh1, Mdh2, Suclg1, Suclg2
WT	Metabolic pathways	1.36e−08	Abat, Acaa2, Acat1, Aco2, Adssl1, Akr1a1, Akr1b10, Aldh3a2, Aldh6a1, Aldoa, Atic, Atp5a1, Atp5c1, Atp5j, Atp6v1e1, Atp6v1f, Atp6v1g1, Cmas, Cndp2, Dlat, Dld, Gba, Glud1, Hexa, Hexb, Hk1, Hprt, Idh2, Isyna1, Ivd, Ldha, Lpcat2, Lta4h, Mogs, Ndufa4, Ndufv1, Pccb, Pdhb, Pgam1, Pon3, Ptgs1, Sdha, Uqcrc1, Uqcrc2, Uqcrfs1
KO		4.54e−08	Ak2, Aldoa, Alox5, Apip, Atp6v1f, Atp6v1g1, Cox5a, Cryl1, Echs1, Eno2, Gatm, Got1, Hexa, Hexb, Ldhb, Lta4h, Mdh1, Mdh2, Ndufb4, Pnp, Ppt1, Suclg1, Suclg2, mt-Co2
WT	Lysosome	4.24e−08	Cd68, Ctsa, Ctsb, Ctsf, Ctsl, Fuca1, Gba, Hexa, Hexb, Lamp1, Lamp2, Npc2, Pla2g15, Psap
KO		4.15e−08	Cd68, Ctsb, Ctsl, Ctsz, Hexa, Hexb, Lamp2, M6pr, Npc2, Ppt1
WT	Oxidative phosphorylation	6.33e−07	Atp5a1, Atp5c1, Atp5j, Atp6v1e1, Atp6v1f, Atp6v1g1, Cox7a2, Ndufa4, Ndufv1, Sdha, Uqcrc1, Uqcrc2, Uqcrfs1
KO		0.0073	Atp6v1f, Atp6v1g1, Cox5a, Ndufb4, mt-Co2
WT	Carbon metabolism	6.33e−07	Acat1, Aco2, Aldh6a1, Aldoa, Dlat, Dld, Hk1, Idh2, Pccb, Pdhb, Pgam1, Sdha
KO		2.15e−06	Aldoa, Echs1, Eno2, Got1, Mdh1, Mdh2, Suclg1, Suclg2
WT	Parkinson s disease	1.23e−06	Atp5a1, Atp5c1, Atp5j, Cox7a2, Ndufa4, Ndufv1, Sdha, Slc25a5, Uba1, Uqcrc1, Uqcrc2, Uqcrfs1, Vdac1
KO		0.0094	Cox5a, Ndufb4, Vdac1, Vdac2, mt-Co2
WT	Pyruvate metabolism	1.92e−06	Acat1, Acyp1, Akr1b10, Aldh3a2, Dlat, Dld, Ldha, Pdhb
KO		0.12	Ldhb, Mdh1, Mdh2
WT	Glycolysis / Gluconeogenesis	2.95e−06	Akr1a1, Aldh3a2, Aldoa, Dlat, Dld, Hk1, Ldha, Pdhb, Pgam1
KO		0.29	Aldoa, Eno2, Ldhb
WT	Huntington disease	1.17e−05	Atp5a1, Atp5c1, Atp5j, Cox7a2, Ndufa4, Ndufv1, Sdha, Slc25a5, Sod1, Uqcrc1, Uqcrc2, Uqcrfs1, Vdac1
KO		0.15	Cox5a, Ndufb4, Vdac1, Vdac2, mt-Co2
WT	Citrate cycle (TCA cycle)	4.85e−05	Aco2, Dlat, Dld, Idh2, Pdhb, Sdha
KO		0.00035	Mdh1, Mdh2, Suclg1, Suclg2
WT	Propanoate metabolism	4.85e−05	Abat, Acat1, Aldh3a2, Aldh6a1, Ldha, Pccb
KO		0.00035	Echs1, Ldhb, Suclg1, Suclg2
WT	Phagosome	0.00077	Atp6v1e1, Atp6v1f, Atp6v1g1, C3, Canx, Ctsl, Itgam, Lamp1, Lamp2, Sec22b
KO		0.00033	Atp6v1f, Atp6v1g1, Ctsl, Lamp2, M6pr, Stx7, Tuba4a
WT	Other glycan degradation	0.0011	Fuca1, Gba, Hexa, Hexb
KO		0.30	Hexa, Hexb

### Expression of miR-146a and Its Target Genes in Brain White Matter Lesions of MS Patients

**Figure 10:**
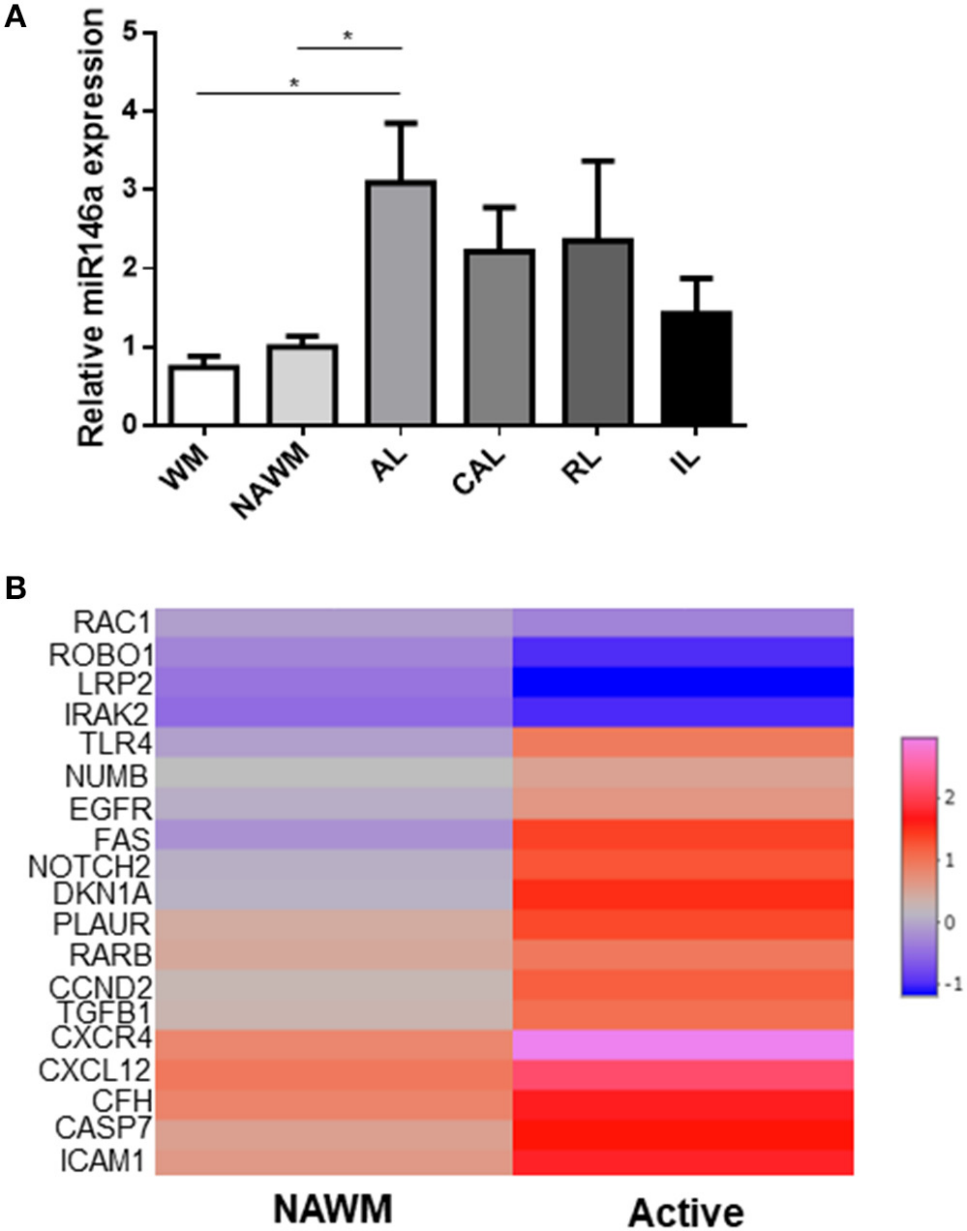
Expression of miR-146a and its target genes in different stages of lesion evolution in the brain white matter of MS patients. **(A)** The expression level of miR-146a was examined by qPCR in microdissected lesions representing different stages of lesion evolution in the MS brain. *p < 0.05, n=4–9, one-way ANOVA followed by Tukey's *post hoc* test, mean ± SD. **(B)** The 88 validated target genes of miR-146a were retracted from the miRTarBase, and their RNA expression level was examined in the same lesions by using the MS-Atlas (www.msatlas.dk). Nineteen genes were significantly differentially regulated in active lesions compared to control. NAWM, normal-appearing white matter; AL, active lesion; CAL, chronic active lesion; RL, remyelinating lesion.

Differential expression of miR-146a has been described in MS lesions examined by microarray (25). Here, we examined the relative expression of miR-146a by qPCR in different lesion types of the brain white matter (Figure 10A). We found the highest expression in active lesions, although it was also expressed in the normal-appearing white matter (NAWM), chronic active as well as remyelinating lesions. We also examined the absolute expression of validated target genes of miR-146a (miRTarBase) determined by RNA sequencing in the active lesions with the highest expression of miR-146a (www.msatlas.dk) (Figure 10B). Nineteen of 88 strongly validated target genes were significantly changed in active lesions (Figure 10B), while none were changed in NAWM compared to control (data not shown). We also examined the differential regulation of these targets in the microglia proteomes, but only MIF was identified.

## Discussion

In this study, we quantified and compared the expression of miR-146a among resting CNS resident cell types *in vitro*, and found the highest expression in microglia. Previous microarray data (41) and data obtained from cell cultures (42) also pointed to an enriched expression of miR-146a in microglia compared to other CNS resident cells. LPS induced an upregulation of miR-146a in BV-2 and EOC 13.31 microglial cell lines (43), as well as in primary microglia cells (44). Based on these results, and since miR-146a is also known to be involved in macrophage cell proliferation (26), we first examined if the absence of miR-146a alters the level of microglia in the adult mouse brain. We did not find any difference in the number of microglia between miR-146a KO mice and WT mice, which indicates that the level of microglia in the brain is not affected by miR-146a deficiency.

We next examined the functional role of miR-146a in microglia cells in response to LPS stimulation *in vitro* by measuring the level of cytokines and chemokines secreted by stimulated miR-146a KO and WT microglia. As expected, we found that microglia expressed various cytokines and chemokines in response to LPS stimulation, but the expression level was significantly higher in microglia lacking miR-146a. This is in line with the suggested role of miR-146a in macrophages as a regulator of the inflammatory response (10), and the findings of others showing that miR-146a suppressed the inflammatory response to LPS in macrophages/monocytes (45, 46). Since miR-146a plays an important role in response to myelin damage *in vivo* (16), and exposure to myelin *in vitro* has been found to induce a unique pattern of cytokine and chemokine production in microglia (47), we examined microglia response to myelin stimulation *in vitro*. We found that myelin-stimulated miR-146a KO microglia cells produced higher levels of TNF, IL-1b, IL-6, IL-10, and CCL3 compared to WT cells. Despite increased expression of cytokine/chemokines in the absence of miR-146a in microglia, no specific pattern was observed: both pro- and anti-inflammatory cytokines were increased.

Several previous studies examined microglia cytokine and chemokine responses during *in viv*o demyelination and remyelination in the CPZ model. Demyelination induced increased phagocytosis by microglia accompanied by upregulation of corresponding receptors and shift in microglia phenotype: intracellular TNF production was increased, while anti-inflammatory IL-10, IL-12, and pro-inflammatory IL-12 and IFN-γ production was not altered (22). Gene and protein expression of two chemokines involved in MS pathology, CCL2 (MCP1) and CCL3 (MIP-1a) were induced by demyelination although with different kinetics (16, 21, 48), and here we also found increased production of both chemokines in microglia stimulated by LPS *in vitro*. The gene of CXCL10 was also upregulated during early remyelination in the corpus callosum, and it may be produced by astrocytes and orchestrate early microglia activation (24, 49). CXCR3, the receptor of CXCL10 is critical for the activation of microglia, and proinflammatory transcripts were attenuated in the brain of CXCR3-deficient mice during CPZ-induced demyelination (50). Another chemokine, CXCL12 was also found to be increased and expressed by CD11b^+^ microglia after 6-week demyelination along with its receptor CXCR4 on OPCs, and maybe involved in promoting remyelination (51). In our previous microarray analysis, a number of genes related to microglia function were differentially expressed in the demyelinating and remyelinating corpus callosum with different kinetics (24): among them, genes of several molecules involved in phagocytosis (CD14, FCGR1, FCGR2, IFGP2, TIM3, ITB2, GAL3); the phagocytic receptor TREM2 was upregulated during early remyelination supporting previous observations (22). IL-6 produced by astrocytes may regulate expression of TREM2 during experimental demyelination and reduce microglia activation and proliferation in connection with reduced demyelination (52). However, we found decreased amount of IL-6 in the miR-146 KO mice during demyelination, while these mice were protected against myelin loss (16). Here we observed that absence of miR-146 increased the production of IL-6 in response to both LPS and myelin, indicating that cytokine and chemokine changes within the tissue are the results of complex interaction among different cells and their functional changes. Transgenic production of IL-17 by astrocytes increased the accumulation of activated microglia in the demyelinating corpus callosum, but the expression of TNF, CCL2, CCL3, and CXCL10 genes was not different compared to WT mice (53). Immunization with MOG peptide, i.e., induction of EAE during CPZ-induced demyelination resulted in microglia activation in contrast to classic EAE, and also potentiated gene expression of CXCL10, CCL2, and CCL3 in the corpus callosum beside additional brain areas (54). Pretreatment of mice with glatiramer acetate (GA), an approved medication of relapsing MS induced microglia activation during demyelination, and microglia cultures treated with GA produced IL-4 and IL-10 (55).

Besides the secretion of cytokines and chemokines, we also examined if absence of miR-146a alters phagocytosis and migration capacity. We found that the phagocytotic ability of LPS stimulated microglia decreased in the absence of miR-146a. This is in line with studies concerning monocytes and macrophages suggesting that an overexpression of miR-146a is associated with increased phagocytosis while miR-146a inhibition is associated with decreased phagocytosis (56, 57). We have recently found a reduced number of microglia in the corpus callosum of miR-146a KO mice during CPZ-induced demyelination (16), which suggest a reduced migration of microglia in the KO mice. In line with those findings, we observed that the absence of miR-146a was associated with decreased migration of myelin and LPS activated microglia when compared to WT controls. Altogether, this indicates that miR-146a plays a main role in activated microglia where it helps induce a migrating and phagocytosing phenotype while dampening cytokine and chemokine production. This may contribute to the reduced demyelination induced by CPZ in miR-146a KO mice (16).

To investigate the role of miR-146a in microglia activation during demyelination, we used the CPZ mouse model of experimental de- and remyelination (7, 21, 22). CD11c^+^ microglia cells are considered effective in presenting antigens, but express proinflammatory cytokines at a lower level than CD11c^−^ microglia (7, 8, 40). We found that the percentage of CD11c^+^ microglia increased in response to CPZ treatment in the WT mice, which is in line with previous findings (7). However, the increase was lower in the miR-146a KO mice compared to WT mice. Others have suggested that a reduced phenotype shift toward CD11c^+^ phenotype in response to CPZ exposure is linked to impaired repair mechanisms (23), but since we have previously shown that miR-146a KO mice were protected against CPZ induced demyelination, axonal loss and weight loss (16), it is likely that the difference in percentage of CD11c^+^ microglia observed in the present study is linked to the reduced demyelination damage of the miR-146a KO mice.

Next, we sorted out resting microglia from WT and KO mice, and also *in vivo* activated microglia from CPZ-exposed WT and KO mice, and conducted a label free quantitative proteomics analysis by mass spectrometry. In resting microglia, we found no significant difference in the proteome between WT and KO mice. During CPZ-induced demyelination, using a stringent threshold level of FDR <0.05, we identified 137 dysregulated proteins in WT microglia, whereas only 10 proteins were dysregulated in miR-146a KO microglia. The lower level of dysregulated proteins in the KO microglia can be related to a lower demyelination damage observed in these mice (16), or to a less changed phenotype caused by the absence of miR-146. We identified 4 out of the 10 changed proteins to be specifically regulated in miR-146a KO microglia. These were GOT1, COX5B, CRYL1, and CST3 proteins. GOT1, which was upregulated in the miR-146a KO microglia, is a transaminase enzyme, which has previously been shown to be neuroprotective in animal models of amyotrophic lateral sclerosis (ALS) and cerebral ischemia, regulating the level of extracellular glutamate (58, 59). COX5B was also upregulated in the miR-146a KO microglia. This protein is a mitochondrial membrane protein found in all cell types participating in the synthesis of ATP. In neuronal cultures COX5B has been reported to be neuroprotective in response to oxygen-glucose deprivation (OGD) (60), and in MS lesions, the gene coding for COX5b is downregulated (61). CST3 was downregulated in miR-146a KO microglia. It is an inhibitor of lysosomal proteinases, and upregulation of CST3 in glial cells has been implicated in the process of neuronal death (62). On the contrary, it has been suggested that CST3 is neuroprotective in Alzheimer's disease (63, 64). CRYL1 was also downregulated in miR-146a KO microglia. This protein is involved in energy production by catalyzing the dehydrogenation of L-gulonate in the alternative glucose metabolic pathway, the uronate cycle (65). To our knowledge, the potential implication of CRYL1 in brain disorders has not been examined. Mitochondrial injury and oxidative stress are believed to be fundamental elements of CPZ induced pathology (66). In addition, activation of microglia in response to myelin internalization is linked to the stimulation of repair processes in response to tissue damage in MS (67). Based on the known function of the changed proteins as described above, it is possible that the upregulation of GOT1 and COX5b, and downregulation of CST3 and CRYL1 in miR-146a KO microglial cells is involved in protecting the miR-146a KO mice from demyelination in response to CPZ exposure (16) by reducing oxidative damage and rendering a repair prone microglia phenotype. However, this hypothesis needs to be further addressed. For STRING analysis of enriched KEGG pathways, we used a more relaxed statistics (FDR 0.15), thus pathway analyses of WT microglia and miR-146a KO microglia included 320 and 101 dysregulated proteins in response to CPZ-induced demyelination, respectively. We identified 14 pathways that were enhanced in both WT and KO microglia from CPZ-treated mice indicating that they are independent of miR-146a. We also found 16 pathways that were only enhanced in WT microglia indicating an indirect regulation by miR-146a. Among these were pathways for Alzheimer disease, antigen processing and presentation and fatty acid degradation indicating a role for miR-146a in these processes. Two pathways, the cysteine/methionine metabolism pathway and the cGMP-PKG signaling pathway were specifically enriched in miR-146a KO microglia indicating direct regulation by miR-146a. However, only 2 proteins in these pathways were not found in the WT proteomics dataset, therefore, enrichment of these pathways in the KO microglia can be a statistical artifact due to a lower total number of changed proteins in the miR-146a KO microglia.

Besides directly comparing the dysregulated proteins in *ex vivo* microglia among the different *in vivo* conditions i.e., in the WT and KO mice with or without CPZ treatment, we further explored discriminative features of microglia proteomes by Principle Component Analysis (PCA) and Partial Least Squares-Discriminant Analysis (sPLS-DA). Both showed that discrimination among the microglia proteomes was the best when control and CPZ-treated conditions were compared. Control WT microglia were separated from the other three groups (control KO, CPZ-treated WT and KO) by 50 proteins based on sPLS-DA, and seven proteins were able to separate microglia according to proteome with or without CPZ treatment. The STRING analysis of these proteins showed significant functional interactions based on gene ontologies (GO) related to ganglioside catabolic and metabolic process, lysosome organization, lipid storage, carbohydrate derivative metabolic process indicating myelin pathways related probably to myelin phagocytosis. However, significant interactions were also related to mRNA metabolic process, processing, and splicing, but also biological processes related to negative regulation of amine transport, cellular protein localization and negative regulation of catecholamine secretion. These data indicate that microglia respond to CPZ by dysregulation of several pathways that are related not only to demyelination but also to the cellular stress. Similar stress responses have also been recently found by examination of the proteome and modified proteome of bulk corpus callosum tissue during CPZ-induced de- and remyelination (68). It is unclear, if such CPZ-induced stress responses that may be different in oligodendrocytes are responsible for preventing microglia apoptosis while inducing death of other cell types, such as oligodendrocytes and thymocytes in this model (69, 70). Finally, cluster of ten proteins separated wild-type and KO microglia after 4-week CPZ treatment based on sPLS-DA, and they represented pathways of plasma membrane organization, synaptic vesicle recycling, vesicle-mediated transport in synapse and neurotransmitter secretion. Among these 10 proteins, NGP (neutrophilic granule protein) that differentiate microglia from peripheral macrophages is a “sensome' allowing cells to perceive and interact with the environment; another differentially expressed protein Hexb also belongs to this group (6). One of the proteins in this discriminating cluster, S100A9 up-regulated IL-1beta, TNF and iNOS in microglia cell line (71). Additional proteins related to synapse and vesicle organization may reflect differential phagocytotic ability of WT and KO microglia (Syn2, Rab3a, Sh3gl2, Clic1). Atp1a3 that belongs to the signature of CD11c^+^ microglia (72) was also found among the cluster proteins, supporting our data that CPZ induces this microglia phenotype. Dysregulated proteins such as Ppm1g may indicate differential stress responses between WT and KO microglia after CPZ treatment, and this protein was recently identified in the proteome of microglia cell line activated with LPS/IFN-γ (73).

Finally, by analyzing different WM lesions in the brain of patients with progressive MS, we found that while miR-146 mRNA was increased in active lesions, it was not significantly higher in NAWM, inactive, chronic active, and remyelinating lesions, supporting previous microarray data that indicated expression in the active but not in inactive lesions (25). We therefore searched for target genes of miR-146a in the same lesions by using the recently established MS-Atlas (www.msatlas.dk). We found that out of 88 target genes, 19 were significantly expressed in active lesions but none in the NAWM. Four target genes among them *IRAK2* were downregulated in active lesions. Fifteen out of the 19 target genes were upregulated, among them the genes of the receptor-ligand pair CXCR4 and CXCL12 that promotes differentiation of oligodendrocyte progenitors and remyelination (51). Genes of cell death and fate (*FAS, CASP7, EGFR*), inflammatory pathway molecules (*ICAM1, TGFB1)* and major target pathways of miR-146a *(TLR4, NOTCH2, NUMB*) were also upregulated.

In summary, here we identified microglia as the main cellular source of miR-146a in the resting brain and aimed at characterizing the phenotype of miR-146a KO microglia. We found that microglia cells are highly activated by LPS and myelin stimulation *in vitro* in the absence of miR-146a. *In vivo*, the CD11c^+^ phenotype associated with antigen presentation is decreased in the KO microglia, which may be explained by less myelin damage in the KO mice (16). In addition, the number of dysregulated proteins is also less in the KO microglia proteome after *in vivo* activation with CPZ. We further identified GOT1, COX5B, CRYL1, and CST3 proteins to be specifically regulated in miR-146a KO microglia in response to CPZ exposure *in vivo*, and hypothesize that the differential regulation of these proteins could be involved in the induction of a microglia phenotype that may contribute to protection against demyelination and axonal loss in the miR-146a KO mice (16). Our data also indicate that miR-146a expression is increased in active lesions of the MS brain, and several target genes are also differentially regulated. These data indicate a heterogeneous role of miR-146a in microglia that may contribute to different pathological outcomes in mouse and potentially in the MS brain depending on the inflammatory environment.

## Data Availability Statement

The datasets generated for this study can be found in the PRIDE repository with the dataset identifier PXD015939.

## Ethics Statement

The studies involving human participants were reviewed and approved by UK Multicentre Research Ethics Committee, MREC/02/2/39. The patients/participants provided their written informed consent to participate in this study. The animal study was reviewed and approved by Danish Animal Health Care Committee (Approval No: 2014-15-00369).

## Author Contributions

NM: conception or design of the work, acquisition, analysis or interpretation of data, drafting the work. KH and ME: acquisition, analysis or interpretation of data, drafting the work. ET, AW, KE, CA, JO, EB, RR, and ZH: acquisition, analysis, or interpretation of data. AS: analysis or interpretation of data, revising it critically for important intellectual content. ÅFS: conception or design of the work, analysis or interpretation of data, revising it critically for important intellectual content. TO: conception or design of the work, analysis or interpretation of data, revising it critically for important intellectual content. ZI: conception or design of the work, analysis or interpretation of data, revising it critically for important intellectual content, drafting the work.

## Conflict of Interest

The authors declare that the research was conducted in the absence of any commercial or financial relationships that could be construed as a potential conflict of interest.

## References

[1] Colonna M Butovsky O. Microglia function in the central nervous system during health and neurodegeneration. Ann Rev Immunol. (2017) 35:441–68. 10.1146/annurev-immunol-051116-052358 28226226 PMC8167938

[2] Luo C Jian C Liao Y Huang Q Wu Y Liu X. The role of microglia in multiple sclerosis. Neuropsychiatr Dis Treat. (2017) 13:1661–7. 10.2147/NDT.S140634 28721047 PMC5499932

[3] Loane DJ Kumar A. Microglia in the TBI brain: the good, the bad, and the dysregulated. Exp Neurol. (2016) 275:316–27. 10.1016/j.expneurol.2015.08.018 26342753 PMC4689601

[4] Lannes N Eppler E Etemad S Yotovski P Filgueira L. Microglia at center stage: a comprehensive review about the versatile and unique residential macrophages of the central nervous system. Oncotarget. (2017) 8:114393–413. 10.18632/oncotarget.23106 29371994 PMC5768411

[5] Tang Y Le W. Differential roles of M1 and M2 microglia in neurodegenerative diseases. Mol Neurobiol. (2016) 53:1181–94. 10.1007/s12035-014-9070-5 25598354

[6] Orihuela R McPherson CA Harry GJ. Microglial M1/M2 polarization and metabolic states. Br J Pharmacol. (2016) 173:649–65. 10.1111/bph.13139 25800044 PMC4742299

[7] Remington LT Babcock AA Zehntner SP Owens T. Microglial recruitment, activation, and proliferation in response to primary demyelination. Am J Pathol. (2007) 170:1713–24. 10.2353/ajpath.2007.060783 17456776 PMC1854965

[8] Wlodarczyk A Lobner M Cedile O Owens T. Comparison of microglia and infiltrating CD11c(+) cells as antigen presenting cells for T cell proliferation and cytokine response. J Neuroinflam. (2014) 11:57. 10.1186/1742-2094-11-57 24666681 PMC3987647

[9] O'Brien J Hayder H Zayed Y Peng C. Overview of MicroRNA biogenesis, mechanisms of actions, and circulation. Front Endocrinol. (2018) 9:402. 10.3389/fendo.2018.00402 30123182 PMC6085463

[10] Saba R Sorensen DL Booth SA. MicroRNA-146a: a dominant, negative regulator of the innate immune response. Front Immunol. (2014) 5:578. 10.3389/fimmu.2014.00578 25484882 PMC4240164

[11] Liu XS Chopp M Pan WL Wang XL Fan BY Zhang Y. MicroRNA-146a promotes oligodendrogenesis in stroke. Mol Neurobiol. (2017) 54:227–37. 10.1007/s12035-015-9655-7 26738853 PMC4935640

[12] Taganov KD Boldin MP Chang KJ Baltimore D. NF-kappaB-dependent induction of microRNA miR-146, an inhibitor targeted to signaling proteins of innate immune responses. Proc Natl Acad Sci USA. (2006) 103:12481–6. 10.1073/pnas.0605298103 16885212 PMC1567904

[13] Kumar H Kawai T Akira S. Pathogen recognition by the innate immune system. Int Rev Immunol. (2011) 30:16–34. 10.3109/08830185.2010.529976 21235323

[14] Israel A. The IKK complex, a central regulator of NF-kappaB activation. Cold Spring Harb Perspect Biol. (2010) 2:a000158. 10.1101/cshperspect.a000158 20300203 PMC2829958

[15] Quinn EM Wang JH O'Callaghan G Redmond HP. MicroRNA-146a is upregulated by and negatively regulates TLR2 signaling. PLoS One. (2013) 8:e62232. 10.1371/journal.pone.0062232 23638011 PMC3639252

[16] Martin NA Molnar V Szilagyi GT Elkjaer ML Nawrocki A Okarmus J. Experimental demyelination and axonal loss are reduced in MicroRNA-146a deficient mice. Front Immunol. (2018) 9:490. 10.3389/fimmu.2018.00490 29593734 PMC5857529

[17] Zhang J Zhang ZG Lu M Wang X Shang X Elias SB. MiR-146a promotes remyelination in a cuprizone model of demyelinating injury. Neuroscience. (2017) 348:252–63. 10.1016/j.neuroscience.2017.02.029 28237816

[18] Li B Wang X Choi IY Wang YC Liu S Pham AT. miR-146a modulates autoreactive Th17 cell differentiation and regulates organ-specific autoimmunity. J Clin Invest. (2017) 127:3702–16. 10.1172/JCI94012 28872459 PMC5617680

[19] Kipp M Nyamoya S Hochstrasser T Amor S. Multiple sclerosis animal models: a clinical and histopathological perspective. Brain pathol. (2017) 27:123–37. 10.1111/bpa.12454 27792289 PMC8029141

[20] Hiremath MM Saito Y Knapp GW Ting JP Suzuki K Matsushima GK. Microglial/macrophage accumulation during cuprizone-induced demyelination in C57BL/6 mice. J Neuroimmunol. (1998) 92:38–49. 10.1016/S0165-5728(98)00168-4 9916878

[21] Biancotti JC Kumar S de Vellis J. Activation of inflammatory response by a combination of growth factors in cuprizone-induced demyelinated brain leads to myelin repair. Neurochem Res. (2008) 33:2615–28. 10.1007/s11064-008-9792-8 18661234

[22] Voss EV Skuljec J Gudi V Skripuletz T Pul R Trebst C. Characterisation of microglia during de- and remyelination: can they create a repair promoting environment? Neurobiol Dis. (2012) 45:519–28. 10.1016/j.nbd.2011.09.008 21971527

[23] Lampron A Larochelle A Laflamme N Prefontaine P Plante MM Sanchez MG. Inefficient clearance of myelin debris by microglia impairs remyelinating processes. J Exp Med. (2015) 212:481–95. 10.1084/jem.20141656 25779633 PMC4387282

[24] Martin NA Nawrocki A Molnar V Elkjaer ML Thygesen EK Palkovits M. Orthologous proteins of experimental de- and remyelination are differentially regulated in the CSF proteome of multiple sclerosis subtypes. PLoS ONE. (2018) 13:e0202530. 10.1371/journal.pone.0202530 30114292 PMC6095600

[25] Junker A Krumbholz M Eisele S Mohan H Augstein F Bittner R. MicroRNA profiling of multiple sclerosis lesions identifies modulators of the regulatory protein CD47. Brain J Neurol. (2009) 132:3342–52. 10.1093/brain/awp300 19952055

[26] Boldin MP Taganov KD Rao DS Yang L Zhao JL Kalwani M. miR-146a is a significant brake on autoimmunity, myeloproliferation, and cancer in mice. J Exp Med. (2011) 208:1189–201. 10.1084/jem.20101823 21555486 PMC3173243

[27] McCarthy KD de Vellis J. Preparation of separate astroglial and oligodendroglial cell cultures from rat cerebral tissue. J Cell Biol. (1980) 85:890–902. 10.1083/jcb.85.3.890 6248568 PMC2111442

[28] Meyer MK Andersen M Bennike TB Birkelund S Andersen GN Stensballe A Effect of IL-6R inhibition with tocilizumab on the proteome of peripheral blood mononuclear cells from a rheumatoid arthritis patient. J Proteomics Bioinform. (2015) 8:274–82. 10.4172/jpb.1000380

[29] Rudolph JD Cox J. A network module for the perseus software for computational proteomics facilitates proteome interaction graph analysis. J Proteome Res. (2019) 18:2052–64. 10.1021/acs.jproteome.8b00927 30931570 PMC6578358

[30] Rohart F Gautier B Singh A Le Cao KA. mixOmics: an R package for 'omics feature selection and multiple data integration. PLoS Comput Biol. (2017) 13:e1005752. 10.1371/journal.pcbi.1005752 29099853 PMC5687754

[31] Szklarczyk D Gable AL Lyon D Junge A Wyder S Huerta-Cepas J. STRING v11: protein-protein association networks with increased coverage, supporting functional discovery in genome-wide experimental datasets. Nucleic Acids Res. (2019) 47:D607–13. 10.1093/nar/gky1131 30476243 PMC6323986

[32] Vizcaino JA Csordas A del-Toro N Dianes JA Griss J Lavidas I 2016 update of the PRIDE database and its related tools. Nucleic Acids Res. (2016) 44:D447–56. 10.1093/nar/gkv1145 26527722 PMC4702828

[33] Deutsch EW Csordas A Sun Z Jarnuczak A Perez-Riverol Y Ternent T. The proteomeXchange consortium in 2017: supporting the cultural change in proteomics public data deposition. Nucleic Acids Res. (2017) 45:D1100–6. 10.1093/nar/gkw936 27924013 PMC5210636

[34] Martin NA Bonner H Elkjaer ML D'Orsi B Chen G Konig HG BID mediates oxygen-glucose deprivation-induced neuronal injury in organotypic hippocampal slice cultures and modulates tissue inflammation in a transient focal cerebral ischemia model without changing lesion volume. Front Cell Neurosci. (2016) 10:14 10.3389/fncel.2016.00014 26869884 PMC4737886

[35] Stangel M Joly E Scolding NJ Compston DA. Normal polyclonal immunoglobulins ('IVIg') inhibit microglial phagocytosis *in vitro*. J Neuroimmunol. (2000) 106:137–44. 10.1016/S0165-5728(00)00210-1 10814791

[36] Tinevez J-Y Perry N Schindelin J Hoopes GM Reynolds GD Laplantine E. TrackMate: an open and extensible platform for single-particle tracking. Methods. (2017) 115:80–90. 10.1016/j.ymeth.2016.09.016 27713081

[37] Elkjaer ML Frisch T Reynolds R Kacprowski T Burton M Kruse TA Molecular signature of different lesion types in the brain white matter of patients with progressive multiple sclerosis. Acta Neuropathol Commun. (2019) 7:205 10.1186/s40478-019-0855-7 31829262 PMC6907342

[38] Chou CH Shrestha S Yang CD Chang NW Lin YL Liao KW. miRTarBase update 2018: a resource for experimentally validated microRNA-target interactions. Nucleic Acids Res. (2018) 46:D296–302. 10.1093/nar/gkx1067 29126174 PMC5753222

[39] Hsu SD Lin FM Wu WY Liang C Huang WC Chan WL. miRTarBase: a database curates experimentally validated microRNA-target interactions. Nucleic Acids Res. (2011) 39:D163–9. 10.1093/nar/gkq1107 21071411 PMC3013699

[40] Wlodarczyk A Holtman IR Krueger M Yogev N Bruttger J Khorooshi R. A novel microglial subset plays a key role in myelinogenesis in developing brain. EMBO J. (2017) 36:3292–308. 10.15252/embj.201696056 28963396 PMC5686552

[41] Jovicic A Roshan R Moisoi N Pradervand S Moser R Pillai B. Comprehensive expression analyses of neural cell-type-specific miRNAs identify new determinants of the specification and maintenance of neuronal phenotypes. J Neurosci. (2013) 33:5127–37. 10.1523/JNEUROSCI.0600-12.2013 23516279 PMC6705001

[42] Wang WX Visavadiya NP Pandya JD Nelson PT Sullivan PG Springer JE. Mitochondria-associated microRNAs in rat hippocampus following traumatic brain injury. Exp Neurol. (2015) 265:84–93. 10.1016/j.expneurol.2014.12.018 25562527 PMC4346439

[43] Saba R Gushue S Huzarewich RL Manguiat K Medina S Robertson C. MicroRNA 146a (miR-146a) is over-expressed during prion disease and modulates the innate immune response and the microglial activation state. PLoS ONE. (2012) 7:e30832. 10.1371/journal.pone.0030832 22363497 PMC3281888

[44] Jayadev S Case A Alajajian B Eastman AJ Moller T Garden GA. Presenilin 2 influences miR146 level and activity in microglia. J Neurochem. (2013) 127:592–9. 10.1111/jnc.12400 23952003 PMC4346352

[45] Nahid MA Pauley KM Satoh M Chan EK. miR-146a is critical for endotoxin-induced tolerance: implication in innate immunity. J Biol Chem. (2009) 284:34590–9. 10.1074/jbc.M109.056317 19840932 PMC2787321

[46] Zeng Z Gong H Li Y Jie K Ding C Shao Q. Upregulation of miR-146a contributes to the suppression of inflammatory responses in LPS-induced acute lung injury. Exp lung Res. (2013) 39:275–82. 10.3109/01902148.2013.808285 23848342

[47] van Rossum D Hilbert S Strassenburg S Hanisch UK Bruck W. Myelin-phagocytosing macrophages in isolated sciatic and optic nerves reveal a unique reactive phenotype. Glia. (2008) 56:271–83. 10.1002/glia.20611 18069669

[48] Buschmann JP Berger K Awad H Clarner T Beyer C Kipp M. Inflammatory response and chemokine expression in the white matter corpus callosum and gray matter cortex region during cuprizone-induced demyelination. J Mol Neurosci. (2012) 48:66–76. 10.1007/s12031-012-9773-x 22528463 PMC3413816

[49] Clarner T Janssen K Nellessen L Stangel M Skripuletz T Krauspe B. CXCL10 triggers early microglial activation in the cuprizone model. J Immunol. (2015) 194:3400–13. 10.4049/jimmunol.1401459 25725102

[50] Krauthausen M Saxe S Zimmermann J Emrich M Heneka MT Muller M. CXCR3 modulates glial accumulation and activation in cuprizone-induced demyelination of the central nervous system. J Neuroinflam. (2014) 11:109. 10.1186/1742-2094-11-109 24930935 PMC4096537

[51] Patel JR McCandless EE Dorsey D Klein RS. CXCR4 promotes differentiation of oligodendrocyte progenitors and remyelination. Proc Natl Acad Sci USA. (2010) 107:11062–7. 10.1073/pnas.1006301107 20534485 PMC2890706

[52] Petkovic F Campbell IL Gonzalez B Castellano B. Astrocyte-targeted production of interleukin-6 reduces astroglial and microglial activation in the cuprizone demyelination model: implications for myelin clearance and oligodendrocyte maturation. Glia. (2016) 64:2104–19. 10.1002/glia.23043 27535761

[53] Zimmermann J Emrich M Krauthausen M Saxe S Nitsch L Heneka MT. IL-17A promotes granulocyte infiltration, myelin loss, microglia activation, and behavioral deficits during cuprizone-induced demyelination. Mol Neurobiol. (2018) 55:946–57. 10.1007/s12035-016-0368-3 28084589

[54] Ruther BJ Scheld M Dreymueller D Clarner T Kress E Brandenburg LO. Combination of cuprizone and experimental autoimmune encephalomyelitis to study inflammatory brain lesion formation and progression. Glia. (2017) 65:1900–13. 10.1002/glia.23202 28836302

[55] Rosato Siri MV Badaracco ME Pasquini JM. Glatiramer promotes oligodendroglial cell maturation in a cuprizone-induced demyelination model. Neurochem Int. (2013) 63:10–24. 10.1016/j.neuint.2013.04.008 23619394

[56] Cao Z Yao Q Zhang S. MiR-146a activates WAVE2 expression and enhances phagocytosis in lipopolysaccharide-stimulated RAW264.7 macrophages. Am J Transl Res. (2015) 7:1467–74. 26396677 PMC4568802

[57] Pauley KM Stewart CM Gauna AE Dupre LC Kuklani R Chan AL. Altered miR-146a expression in sjogren's syndrome and its functional role in innate immunity. Eur J Immunol. (2011) 41:2029–39. 10.1002/eji.201040757 21469088 PMC3760391

[58] Perez-Mato M Ramos-Cabrer P Sobrino T Blanco M Ruban A Mirelman D. Human recombinant glutamate oxaloacetate transaminase 1 (GOT1) supplemented with oxaloacetate induces a protective effect after cerebral ischemia. Cell Death Dis. (2014) 5:e992. 10.1038/cddis.2013.507 24407245 PMC4040715

[59] Ruban A Malina KC Cooper I Graubardt N Babakin L Jona G. Combined treatment of an amyotrophic lateral sclerosis rat model with recombinant got1 and oxaloacetic acid: a novel neuroprotective treatment. Neuro Degenerative Dis. (2015) 15:233–42. 10.1159/000382034 26113413

[60] Dai C Liang D Li H Sasaki M Dawson TM Dawson VL. Functional identification of neuroprotective molecules. PLoS ONE. (2010) 5:e15008. 10.1371/journal.pone.0015008 21124846 PMC2991347

[61] Safavizadeh N Rahmani SA Zaefizadeh M. Investigation of cytocrom c oxidase gene subunits expression on the multiple sclerosis. Indian J Hum Gene. (2013) 19:18–25. 10.4103/0971-6866.112879 23901189 PMC3722625

[62] Nagai A Terashima M Sheikh AM Notsu Y Shimode K Yamaguchi S. Involvement of cystatin C in pathophysiology of CNS diseases. Front Biosci. (2008) 13:2941. 10.2741/2941 18508448

[63] Butler JM Sharif U Ali M McKibbin M Thompson JP Gale R. A missense variant in CST3 exerts a recessive effect on susceptibility to age-related macular degeneration resembling its association with Alzheimer's disease. Hum Gene. (2015) 134:705–15. 10.1007/s00439-015-1552-7 25893795 PMC4460273

[64] Kaur G Levy E. Cystatin C in alzheimer's disease. Front Mol Neurosci. (2012) 5:79. 10.3389/fnmol.2012.00079 22783166 PMC3390601

[65] Ishikura S Usami N Araki M Hara A. Structural and functional characterization of rabbit and human L-gulonate 3-dehydrogenase. J Biochem. (2005) 137:303–14. 10.1093/jb/mvi033 15809331

[66] Praet J Guglielmetti C Berneman Z Van der Linden A Ponsaerts P. Cellular and molecular neuropathology of the cuprizone mouse model: clinical relevance for multiple sclerosis. Neurosci Biobehav Rev. (2014) 47:485–505. 10.1016/j.neubiorev.2014.10.004 25445182

[67] Bogie JF Stinissen P Hendriks JJ. Macrophage subsets and microglia in multiple sclerosis. Acta Neuropathol. (2014) 128:191–213. 10.1007/s00401-014-1310-2 24952885

[68] Szilagyi GT Nawrocki AM Eros K Schmidt J Fekete K Elkjaer ML. Proteomic changes during experimental de- and remyelination in the corpus callosum. PLoS ONE. (2020) 15:e0230249. 10.1371/journal.pone.0230249 32272486 PMC7145428

[69] Veto S Acs P Bauer J Lassmann H Berente Z Setalo G. Inhibiting poly(ADP-ribose) polymerase: a potential therapy against oligodendrocyte death. Brain J Neurol. (2010) 133:822–34. 10.1093/brain/awp337 20157013 PMC2964508

[70] Solti I Kvell K Talaber G Veto S Acs P Gallyas F. Thymic atrophy and apoptosis of CD4+CD8+ thymocytes in the cuprizone model of multiple sclerosis. PLoS ONE. (2015) 10:e0129217. 10.1371/journal.pone.0129217 26053248 PMC4460035

[71] Ha TY Chang KA Kim J Kim HS Kim S Chong YH. S100a9 knockdown decreases the memory impairment and the neuropathology in Tg2576 mice, AD animal model. PLoS ONE. (2010) 5:e8840. 10.1371/journal.pone.0008840 20098622 PMC2809116

[72] Benmamar-Badel A Owens T Wlodarczyk A. Protective microglial subset in development, aging, and disease: lessons from transcriptomic studies. Front Immunol. (2020) 11:430. 10.3389/fimmu.2020.00430 32318054 PMC7147523

[73] Mendonca P Taka E Soliman KFA. Proteomic analysis of the effect of the polyphenol pentagalloyl glucose on proteins involved in neurodegenerative diseases in activated BV2 microglial cells. Mol Med Rep. (2019) 20:1736–46. 10.3892/mmr.2019.10400 31257500 PMC6625426

