# Supplementary material for: Absence of miRNA-146a Differentially Alters Microglia Function and Proteome
Source: Front Immunol. 2020 Jun 5;11:1110. doi: 10.3389/fimmu.2020.01110 (PMC7292149; doi:10.3389/fimmu.2020.01110)
Supplement: Supplementary file 1 [file Table_1.docx]

**Supplementary Table 1**

**Dysregulated proteins in WT microglia in response to CPZ exposure *in vivo***

| **Protein** | | **P-value** | **Change** |
| --- | --- | --- | --- |
| A2mp | Alpha-2-macroglobulin-P | 0.002447 | 9.813464 |
| Apoa1 | Apolipoprotein A-I;Proapolipoprotein A-I;Truncated apolipoprotein A-I | 0.004567 | 9.42764 |
| Itih2 | Inter-alpha-trypsin inhibitor heavy chain H2 | 0.008839 | 8.636697 |
| Cep162 | Centrosomal protein of 162 kDa | 0.002379 | 7.59857 |
| Alb | Serum albumin | 0.007594 | 7.553234 |
| Dsg1a;Dsg1b | Desmoglein-1-alpha;Desmoglein-1-beta | 0.028913 | 5.280632 |
| C3 | Complement C3 | 0.006243 | 5.181028 |
| Apoe | Apolipoprotein E | 0.027081 | 4.602615 |
| Myo9b | Unconventional myosin-IXb | 0.029025 | 4.300843 |
| Tmsb4x | Thymosin beta-4;Hematopoietic system regulatory peptide | 0.012036 | 3.703459 |
| Hexa | Beta-hexosaminidase subunit alpha | 0.004814 | 2.981808 |
| Rps10 | 40S ribosomal protein S10 | 0.007381 | 2.559756 |
| Cstb | Cystatin-B | 0.006974 | 2.492023 |
| Cfl1 | Cofilin-1 | 0.001746 | 2.426387 |
| H2-D1;H2-Q7;H2-Q6;H2-Q9;H2-Q8 | H-2 class I histocompatibility antigen, D-B alpha chain;H-2 class I histocompatibility antigen, Q9 alpha chain;H-2 class I histocompatibility antigen, Q8 alpha chain;H-2 class I histocompatibility antigen, Q7 alpha chain | 0.023378 | 2.394939 |
| Hexb | Beta-hexosaminidase subunit beta | 0.01318 | 2.199349 |
| Crkl | Crk-like protein | 0.002799 | 2.198523 |
| Anxa5 | Annexin A5 | 0.001477 | 2.161939 |
| Atp6v1f | V-type proton ATPase subunit F | 0.006551 | 2.018719 |
| Txn | Thioredoxin | 0.000168 | 2.012399 |
| Capg | Macrophage-capping protein | 0.016154 | 1.972633 |
| Atp6v1g1 | V-type proton ATPase subunit G 1 | 0.000634 | 1.948683 |
| Sod1 | Superoxide dismutase [Cu-Zn] | 0.001369 | 1.864072 |
| Eef1b2;Eef1b | Elongation factor 1-beta | 0.004165 | 1.82025 |
| Prdx1 | Peroxiredoxin-1 | 0.014288 | 1.798847 |
| Psme2 | Proteasome activator complex subunit 2 | 0.013651 | 1.771742 |
| Cirbp | Cold-inducible RNA-binding protein | 0.003819 | 1.760771 |
| Rad23b | UV excision repair protein RAD23 homolog B | 0.000149 | 1.736866 |
| Gsn | Gelsolin | 0.005317 | 1.706744 |
| Adssl1 | Adenylosuccinate synthetase isozyme 1 | 0.005175 | 1.700761 |
| Timm13 | Mitochondrial import inner membrane translocase subunit Tim13 | 0.008516 | 1.68959 |
| Ranbp1 | Ran-specific GTPase-activating protein | 0.005717 | 1.675124 |
| Lsp1 | Lymphocyte-specific protein 1 | 0.003697 | 1.673748 |
| Ywhab | 14-3-3 protein beta/alpha;14-3-3 protein beta/alpha, N-terminally processed | 0.008778 | 1.598787 |
| Scpep1 | Retinoid-inducible serine carboxypeptidase | 0.005732 | 1.59803 |
| Ppm1g | Protein phosphatase 1G | 0.004557 | 1.547815 |
| Aldoa | Fructose-bisphosphate aldolase A;Fructose-bisphosphate aldolase | 0.001323 | 1.465051 |
| Atic | Bifunctional purine biosynthesis protein PURH;Phosphoribosylaminoimidazolecarboxamide formyltransferase;IMP cyclohydrolase | 0.001256 | 1.358522 |
| Ewsr1 | RNA-binding protein EWS | 0.001685 | 0.702455 |
| Aldh6a1 | Methylmalonate-semialdehyde dehydrogenase [acylating], mitochondrial | 0.002143 | 0.608693 |
| Atp1b1 | Sodium/potassium-transporting ATPase subunit beta-1 | 0.004056 | 0.60839 |
| Fus | RNA-binding protein FUS | 0.006868 | 0.607439 |
| Sdha | Succinate dehydrogenase [ubiquinone] flavoprotein subunit, mitochondrial | 0.000471 | 0.606293 |
| Mob1b;Mob1a | MOB kinase activator 1A;MOB kinase activator 1B | 0.000325 | 0.585599 |
| Dynll2;BC048507 | Dynein light chain 2, cytoplasmic | 8.88E-05 | 0.57173 |
| Tia1;Tial1 | Nucleolysin TIA-1;Nucleolysin TIAR | 0.008878 | 0.561225 |
| Sfxn3 | Sideroflexin-3 | 0.013133 | 0.560455 |
| Ncbp1 | Nuclear cap-binding protein subunit 1 | 0.0002 | 0.557634 |
| Pura | Transcriptional activator protein Pur-alpha | 0.004768 | 0.552104 |
| Hnrnpd | Heterogeneous nuclear ribonucleoprotein D0 | 0.000158 | 0.548327 |
| Camk2a;Camk2d;Camk2b | Calcium/calmodulin-dependent protein kinase type II subunit alpha;Calcium/calmodulin-dependent protein kinase type II subunit delta;Calcium/calmodulin-dependent protein kinase type II subunit beta | 0.012691 | 0.546361 |
| Rhog | Rho-related GTP-binding protein RhoG | 0.015992 | 0.538707 |
| Ppp1ca | Serine/threonine-protein phosphatase PP1-alpha catalytic subunit | 0.00663 | 0.516329 |
| Ndufa4 | Cytochrome c oxidase subunit NDUFA4 | 0.015933 | 0.512376 |
| Ugt1a7c;Ugt1a6a;Ugt1a10;Ugt1a5;Ugt1a8;Ugt1a6b;Ugt1a9;Ugt1a6;Ugt1a2;Ugt1a1 | UDP-glucuronosyltransferase 1-7C;UDP-glucuronosyltransferase 1-9;UDP-glucuronosyltransferase 1-6;UDP-glucuronosyltransferase 1-2;UDP-glucuronosyltransferase 1-1 | 0.000998 | 0.502175 |
| Aco2 | Aconitate hydratase, mitochondrial | 0.008745 | 0.497758 |
| Sf3a1 | Splicing factor 3A subunit 1 | 0.004607 | 0.497661 |
| Sec13 | Protein SEC13 homolog | 0.013829 | 0.496363 |
| Pccb | Propionyl-CoA carboxylase beta chain, mitochondrial | 0.011084 | 0.495207 |
| Entpd1 | Ectonucleoside triphosphate diphosphohydrolase 1 | 0.019072 | 0.492402 |
| Nono | Non-POU domain-containing octamer-binding protein | 0.010812 | 0.491394 |
| Rab8b;Rab8a;Rab13 | Ras-related protein Rab-8B;Ras-related protein Rab-8A;Ras-related protein Rab-13 | 0.012102 | 0.489264 |
| Cyb5r3 | NADH-cytochrome b5 reductase;NADH-cytochrome b5 reductase 3;NADH-cytochrome b5 reductase 3 membrane-bound form;NADH-cytochrome b5 reductase 3 soluble form | 0.017714 | 0.482597 |
| Supt16;Supt16h | FACT complex subunit SPT16 | 0.008868 | 0.478285 |
| Sf3b3 | Splicing factor 3B subunit 3 | 0.020501 | 0.477627 |
| Khdrbs1 | KH domain-containing, RNA-binding, signal transduction-associated protein 1 | 0.006506 | 0.476686 |
| Lyn | Tyrosine-protein kinase Lyn | 0.01095 | 0.441805 |
| Sec22b | Vesicle-trafficking protein SEC22b | 0.001611 | 0.436176 |
| Ssr3 | Translocon-associated protein subunit gamma | 0.008827 | 0.434326 |
| Dazap1 | DAZ-associated protein 1 | 0.011792 | 0.434137 |
| Methig1;Mettl7a1;Mettl7a2;Mettl7a3 | Methyltransferase Like 7A | 0.007827 | 0.431342 |
| Snd1 | Staphylococcal nuclease domain-containing protein 1 | 0.009473 | 0.428121 |
| Prpf19 | Pre-mRNA-processing factor 19 | 0.018862 | 0.424199 |
| Prpf4 | U4/U6 small nuclear ribonucleoprotein Prp4 | 0.00341 | 0.422652 |
| Wdr82 | WD repeat-containing protein 82 | 0.005708 | 0.420712 |
| Snrnp40 | U5 small nuclear ribonucleoprotein 40 kDa protein | 0.002898 | 0.419766 |
| Ptgs1 | Prostaglandin G/H synthase 1 | 0.020735 | 0.419725 |
| Nsf | Vesicle-fusing ATPase | 0.021722 | 0.419582 |
| Dcps | m7GpppX diphosphatase | 0.01086 | 0.409753 |
| Hnrnpr | Heterogeneous Nuclear Ribonucleoprotein R | 0.005884 | 0.399237 |
| Ngp | Neutrophilic granule protein | 0.009823 | 0.396206 |
| Ctsl | Cathepsin L1;Cathepsin L1 heavy chain;Cathepsin L1 light chain | 0.000493 | 0.391923 |
| Matr3 | Matrin-3 | 0.015533 | 0.391327 |
| Pon3 | Serum paraoxonase/lactonase 3 | 0.003253 | 0.387666 |
| Safb;Safb2 | Scaffold attachment factor B1;Scaffold attachment factor B2 | 0.013425 | 0.383712 |
| Etfa | Electron transfer flavoprotein subunit alpha, mitochondrial | 0.020011 | 0.381897 |
| Sf3a3 | Splicing factor 3A subunit 3 | 0.003334 | 0.380511 |
| Gtf2i | General transcription factor II-I | 0.016679 | 0.379795 |
| Actl6a | Actin-like protein 6A | 0.003147 | 0.379745 |
| Srrt | Serrate RNA effector molecule homolog | 0.004335 | 0.374869 |
| Kpnb1 | Importin subunit beta-1 | 0.027303 | 0.364382 |
| Thrap3 | Thyroid hormone receptor-associated protein 3 | 0.009882 | 0.362234 |
| Smu1 | WD40 repeat-containing protein SMU1;WD40 repeat-containing protein SMU1, N-terminally processed | 0.003212 | 0.361598 |
| Hnrnpul2 | Heterogeneous nuclear ribonucleoprotein U-like protein 2 | 0.016582 | 0.351483 |
| Raver1 | Ribonucleoprotein PTB-binding 1 | 0.01922 | 0.349973 |
| Tufm | Elongation factor Tu, mitochondrial | 0.004231 | 0.336356 |
| Dhx15 | Pre-mRNA-splicing factor ATP-dependent RNA helicase DHX15 | 0.01228 | 0.322278 |
| Rbm39 | RNA-binding protein 39 | 0.003807 | 0.321262 |
| Pspc1 | Paraspeckle component 1 | 0.000792 | 0.316024 |
| Smarcc2 | SWI/SNF complex subunit SMARCC2 | 0.007056 | 0.312573 |
| Tuba1a;Tuba1c;Tuba3a;Tuba8 | Tubulin alpha-1A chain;Tubulin alpha-1C chain;Tubulin alpha-3 chain;Tubulin alpha-8 chain | 0.000448 | 0.305097 |
| Hk1 | Hexokinase;Hexokinase-1 | 0.008955 | 0.301442 |
| Hnrnph3 | Heterogeneous Nuclear Ribonucleoprotein H3 | 0.027648 | 0.294086 |
| Atp5l | ATP synthase subunit g, mitochondrial | 0.007515 | 0.293707 |
| Aldh3a2 | Aldehyde dehydrogenase;Fatty aldehyde dehydrogenase | 0.00114 | 0.283398 |
| Dld | Dihydrolipoyl dehydrogenase, mitochondrial | 0.012484 | 0.282745 |
| Vrk1 | Serine/threonine-protein kinase VRK1 | 0.006671 | 0.282587 |
| S100a9 | Protein S100-A9 | 0.017912 | 0.277782 |
| Ddx17 | Probable ATP-dependent RNA helicase DDX17 | 0.006349 | 0.275939 |
| Elmo1 | Engulfment and cell motility protein 1 | 0.002937 | 0.265639 |
| Ruvbl1 | RuvB-like 1 | 0.00072 | 0.265485 |
| Hnrnpul1 | Heterogeneous nuclear ribonucleoprotein U-like protein 1 | 0.020618 | 0.263992 |
| Ruvbl2 | RuvB-like 2 | 0.033129 | 0.260008 |
| Epb41l2 | Band 4.1-like protein 2 | 0.002884 | 0.257071 |
| Sun2 | SUN domain-containing protein 2 | 0.023029 | 0.256439 |
| Myef2 | Myelin expression factor 2 | 0.001792 | 0.253699 |
| Phb2 | Prohibitin-2 | 0.018892 | 0.252786 |
| Itgam | Integrin alpha-M | 0.007526 | 0.245033 |
| Cmas | N-acylneuraminate cytidylyltransferase | 0.010038 | 0.244925 |
| Thoc2 | THO complex subunit 2 | 0.011231 | 0.242458 |
| Smc3 | Structural maintenance of chromosomes protein 3 | 0.018586 | 0.23789 |
| P2ry12 | P2Y purinoceptor 12 | 0.019048 | 0.233985 |
| Sf3b1 | Splicing factor 3B subunit 1 | 0.003943 | 0.229894 |
| Ilf2 | Interleukin enhancer-binding factor 2 | 0.000377 | 0.221465 |
| Nudt21 | Cleavage and polyadenylation specificity factor subunit 5 | 6.44E-05 | 0.218889 |
| Lpcat2 | Lysophosphatidylcholine acyltransferase 2 | 0.022293 | 0.21855 |
| Hnrnph2 | Heterogeneous nuclear ribonucleoprotein H2 | 0.00468 | 0.217816 |
| Snrnp200 | U5 small nuclear ribonucleoprotein 200 kDa helicase | 0.013273 | 0.209137 |
| Sqrdl | Sulfide:quinone oxidoreductase, mitochondrial | 0.026061 | 0.189402 |
| Hcfc1 | Host cell factor 1;HCF N-terminal chain 1;HCF N-terminal chain 2;HCF N-terminal chain 3;HCF N-terminal chain 4;HCF N-terminal chain 5;HCF N-terminal chain 6;HCF C-terminal chain 1;HCF C-terminal chain 2;HCF C-terminal chain 3;HCF C-terminal chain 4;HCF C-terminal chain 5;HCF C-terminal chain 6 | 0.00078 | 0.189328 |
| Kpna3;Kpna4 | Importin subunit alpha-4;Importin subunit alpha-3 | 0.008288 | 0.174267 |
| Smc1a | Structural maintenance of chromosomes protein 1A | 0.022465 | 0.171853 |
| Cenpv | Centromere protein V | 0.026346 | 0.170859 |
| Gnb2 | Guanine nucleotide-binding protein G(I)/G(S)/G(T) subunit beta-2 | 0.000589 | 0.167874 |
| Prpf8 | Pre-mRNA-processing-splicing factor 8 | 0.016958 | 0.107541 |
| Parp1 | Poly [ADP-ribose] polymerase 1 | 0.021088 | 0.085478 |
