# Supplementary material for: Absence of miRNA-146a Differentially Alters Microglia Function and Proteome
Source: Front Immunol. 2020 Jun 5;11:1110. doi: 10.3389/fimmu.2020.01110 (PMC7292149; doi:10.3389/fimmu.2020.01110)
Supplement: Supplementary file 2 [file Table_2.docx]

**Supplementary Table 2**

**Discriminative proteins for classifying microglia isolated from wild-type and miR-146a mice with and without cuprizone treatment**

| **Component 1^a^** | **Component 2^a^** | **Component 3^a^** |
| --- | --- | --- |
| Aldoa | Rab3a | Cfl1 |
| Hexb | Ngp | Alb |
| Hexa | Sh3gl2 | Psap |
| Cox5b | S100a9 | Tmem33 |
| Lgals3 | Syn2 | Sf3b6 |
| H2-D1 | Atp1a3 | Slc12a2 |
| Cd180 | D10Jhu81e | Tr2a |
|  | Ppm1g | Mthfd1 |
|  | Clic1 | Entpd1 |
|  | Hebp1 | Raver1 |
|  |  | Nop56 |
|  |  | Rhog |
|  |  | Tmem173 |
|  |  | Hnrnpdl |
|  |  | Fcrls |
|  |  | Ctsf |
|  |  | Safb |
|  |  | P2ry12 |
|  |  | Dpp7 |
|  |  | Thoc2 |
|  |  | Acaa2 |
|  |  | Pla2g15 |
|  |  | Tm9sf3 |
|  |  | Arl6ip5 |
|  |  | Hnrnpm |
|  |  | Gm10093 |
|  |  | Ddx5 |
|  |  | Lmna |
|  |  | Ranbp2 |
|  |  | Lmnb2 |
|  |  | Sun2 |
|  |  | Matr3 |
|  |  | Jtgam |
|  |  | Ptgs1 |
|  |  | Nudt21 |
|  |  | Cenpv |
|  |  | Hnrnpul2 |
|  |  | Anxa6 |
|  |  | Vapa |
|  |  | Sec22b |
|  |  | Pon3 |
|  |  | Prpf4 |
|  |  | Numa1 |
|  |  | Sf3b1 |
|  |  | Ruvbl1 |
|  |  | Actl6a |
|  |  | Lmnb1 |
|  |  | Smarcc2 |
|  |  | Prpf8 |
|  |  | Rpn1 |

^a^the supervised model, Partial Least Squares-Discriminant Analysis (sPLS-DA) was used to identify discriminative proteins in the proteome of microglia isolated from wild-type control, wild-type cuprizone, KO-control and KO-cuprizone mice. See Figure 8 (component 1 and 2) and Figure 9 (component 3). The downward order of proteins follows the order on Figure 8B, 8C, and Figure 9B from left to right. Component 1 showed a clustering of microglia according to cuprizone treatment with upregulation in the treated and downregulation in control mice (Figure 8A and B); component 2 clustered wild-type and KO microglia from cuprizone-treated mice by seven proteins that were downregulated in wild-type and upregulated in KO microglia (Figure 8A and 8C); component 3 clustered control wild-type microglia from all the other groups by 47 proteins that were all upregulated in control wild-type microglia (Figure 9A and B).
