# Supplementary material for: Absence of miRNA-146a Differentially Alters Microglia Function and Proteome
Source: Front Immunol. 2020 Jun 5;11:1110. doi: 10.3389/fimmu.2020.01110 (PMC7292149; doi:10.3389/fimmu.2020.01110)
Supplement: Supplementary file 3 [file Image_1.pdf]

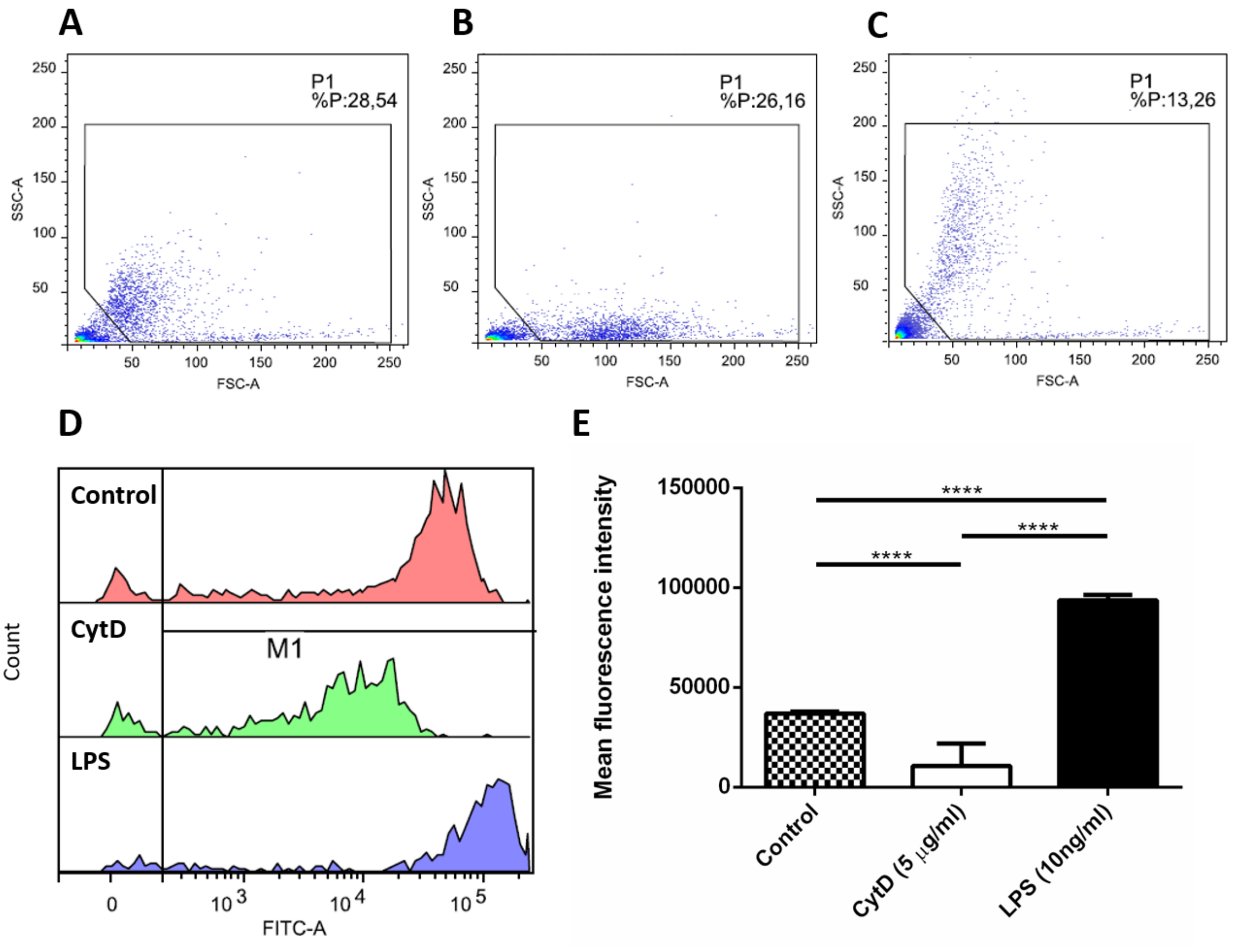

Supplementary Figure 1: Phagocytosis of miR-146a KO and WT microglia treated with LPS and Cytochalasin D. (A) Control microglia (B) Cytochalasin D treated microglia (C) LPS treated microglia (D) Histogram displaying naïve (upper panel) Cytochalasin D treated (middle panel) and LPS treated (lower panel) microglia. (E) Column chart showing phagocytosis of differently treated microglia quantified by mean fluorescent intensity in the FITCH channel. Statistics: **** $p<0.0001$, $n=1355$-1580 in each group, two-way ANOVA followed by Tukey's multiple comparisons test, mean ± 95% confidence interval
